# Polarization Dynamics of Solid-State Quantum Emitters

**DOI:** 10.1021/acsnano.3c08940

**Published:** 2024-02-09

**Authors:** Anand Kumar, Çağlar Samaner, Chanaprom Cholsuk, Tjorben Matthes, Serkan Paçal, Yağız Oyun, Ashkan Zand, Robert J. Chapman, Grégoire Saerens, Rachel Grange, Sujin Suwanna, Serkan Ateş, Tobias Vogl

**Affiliations:** † Department of Computer Engineering, School of Computation, Information and Technology, Technical University of Munich, 80333 Munich, Germany; ‡ Abbe Center of Photonics, Institute of Applied Physics, Friedrich Schiller University Jena, 07745 Jena, Germany; ¶ Department of Physics, İzmir Institute of Technology, 35430 İzmir, Turkey; § Department of Photonics, İzmir Institute of Technology, 35430 İzmir, Turkey; ∥ Optical Nanomaterial Group, Institute for Quantum Electronics, Department of Physics, ETH Zurich, 8093 Zürich, Switzerland; ⊥ Optical and Quantum Physics Laboratory, Department of Physics, Faculty of Science, Mahidol University, 10400 Bangkok, Thailand

**Keywords:** quantum emitters array, hexagonal boron nitride, nanodiamond NV centers, electron irradiation, defect identification, temporal polarization dynamics, density functional theory

## Abstract

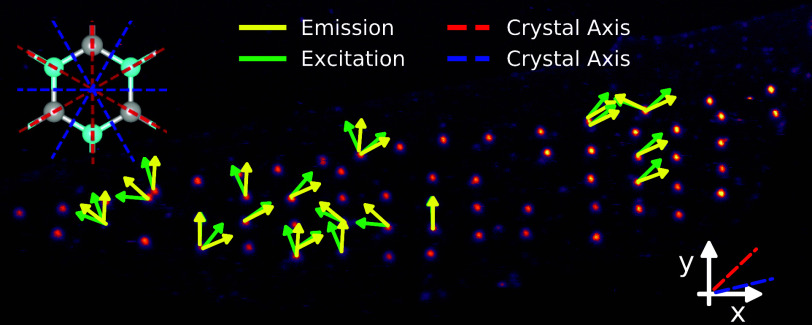

Quantum emitters in solid-state crystals have recently attracted a great deal of attention due to their simple applicability in optical quantum technologies. The polarization of single photons generated by quantum emitters is one of the key parameters that plays a crucial role in various applications, such as quantum computation, which uses the indistinguishability of photons. However, the degree of single-photon polarization is typically quantified using the time-averaged photoluminescence intensity of single emitters, which provides limited information about the dipole properties in solids. In this work, we use single defects in hexagonal boron nitride and nanodiamond as efficient room-temperature single-photon sources to reveal the origin and temporal evolution of the dipole orientation in solid-state quantum emitters. The angles of the excitation and emission dipoles relative to the crystal axes were determined experimentally and then calculated using density functional theory, which resulted in characteristic angles for every specific defect that can be used as an efficient tool for defect identification and understanding their atomic structure. Moreover, the temporal polarization dynamics revealed a strongly modified linear polarization visibility that depends on the excited-state decay time of the individual excitation. This effect can potentially be traced back to the excitation of excess charges in the local crystal environment. Understanding such hidden time-dependent mechanisms can further improve the performance of polarization-sensitive experiments, particularly that for quantum communication with single-photon emitters.

## Introduction

Fluorescent defects in solid-state crystals have become one of the most promising sources of single photons^1^ for near-future quantum information processing and integrated quantum photonics.^2^ The key roles of single-photon sources for optical quantum computing,^3^ quantum key distribution (QKD),^4^ nanoscale quantum sensors,^5^ and fundamental quantum optics experiments^6^ have fueled the research on quantum emitter (QE) systems. Particularly important for these photon sources is the stability of their intrinsic properties. An unsteady polarization, for example, limits the coherence (for interferometry), indistinguishability (for optical quantum computing), or entropy (for QKD) of a photon source. There are many material platforms that have been identified that can host stable single-photon emitters at room temperature, including hexagonal boron nitride (hBN),^7^ diamond,^8^ silicon nitride,^9^ and zinc oxide,^10^ to name only a few. One aspect that all of these systems have in common is that a point-like defect induces additional energy levels into the wide band gap of the host materials.

Due to the relatively recent discovery of fluorescent defects in hBN,^7^ their atomic structures are not well understood compared to established emitter systems,^11^ such as the nitrogen vacancy (NV) centers in diamond.^12^ Defects in hBN can be created artificially via localized electron and ion implantation,^13^ oxygen plasma treatment,^14^ chemical etching,^15^ and γ-rays,^16^ and they can be activated through strain.^17^ It is possible to control the defect formation such that emitter arrays can be fabricated using nanoindentation with an atomic force microscope^18^ and localized electron irradiation.^19−21^

Recent investigations have identified the negatively charged boron vacancy as a near-infrared emitter through optically detected magnetic resonance measurements.^22^ Other experiments have linked carbon impurities to visible emitters in the blue^20^ and green-red^23^ regions of the spectrum. These works have used magnetic or spectral properties for emitter identification. Another option could be to use dipole polarization dynamics, which is also characteristic of every specific defect. This is, however, meaningful only when a large number of identical emitters are investigated and sufficient statistics are collected. The fabrication of such identical emitter arrays has been achieved recently.^19−21^ However, the systematic study of the emission dipole angles has remained inconclusive, as either only a few emitters were studied^19,24^ or the dipoles were randomly distributed.^25^ The latter could indicate a surface complex that does not form a chemical bond with the hBN lattice and, therefore, could be oriented randomly. Vacancy related defects in wrinkled hBN have shown a strong correlation of the polarization axes with the wrinkle direction in the crystal.^26^ Various experimental and theoretical models have advanced the insight into emitters, yet identifying the defects has remained elusive.^19,25,27−29^

When such defect-based emitters are used in quantum communication scenarios and when information is encoded in the polarization, recent studies have demonstrated a performance improvement of the quantum communication protocols by temporal filtering and post-selection.^30,31^ This was always a well-known effect due to detector dark counts that can be suppressed this way. As the emission process of a fluorescent defect is usually complex, it is important to understand the emission dynamics, which can potentially enhance the performance in quantum technology applications even further. There have been some insights into the dynamics of the optical transitions from multiple electronic excited states in hBN,^32^ but the relation to the transition dipole moments is still unknown.

In this work, we study the polarization dynamics of a large array of identical “yellow” quantum emitters. We investigated the correlation of excitation and emission polarization with the host crystal axes. Our experiments are supported by density functional theory (DFT) calculations, which can model the dipole characteristics that are a characteristic fingerprint of any specific defect. Furthermore, we also time-resolved our polarization measurements to gain insight into the emission mechanism. This is generalized to other samples containing quantum emitters, including hBN nanoflakes and NV centers in diamonds. We, therefore, provide important insights into the polarization dynamics of general solid-state quantum emitters. This includes the oddity of misaligned excitation and emission dipoles, non-unity polarization visibility, as well as the atomic structure of the yellow hBN emitter.

## Results and Discussion

**Figure 1:**
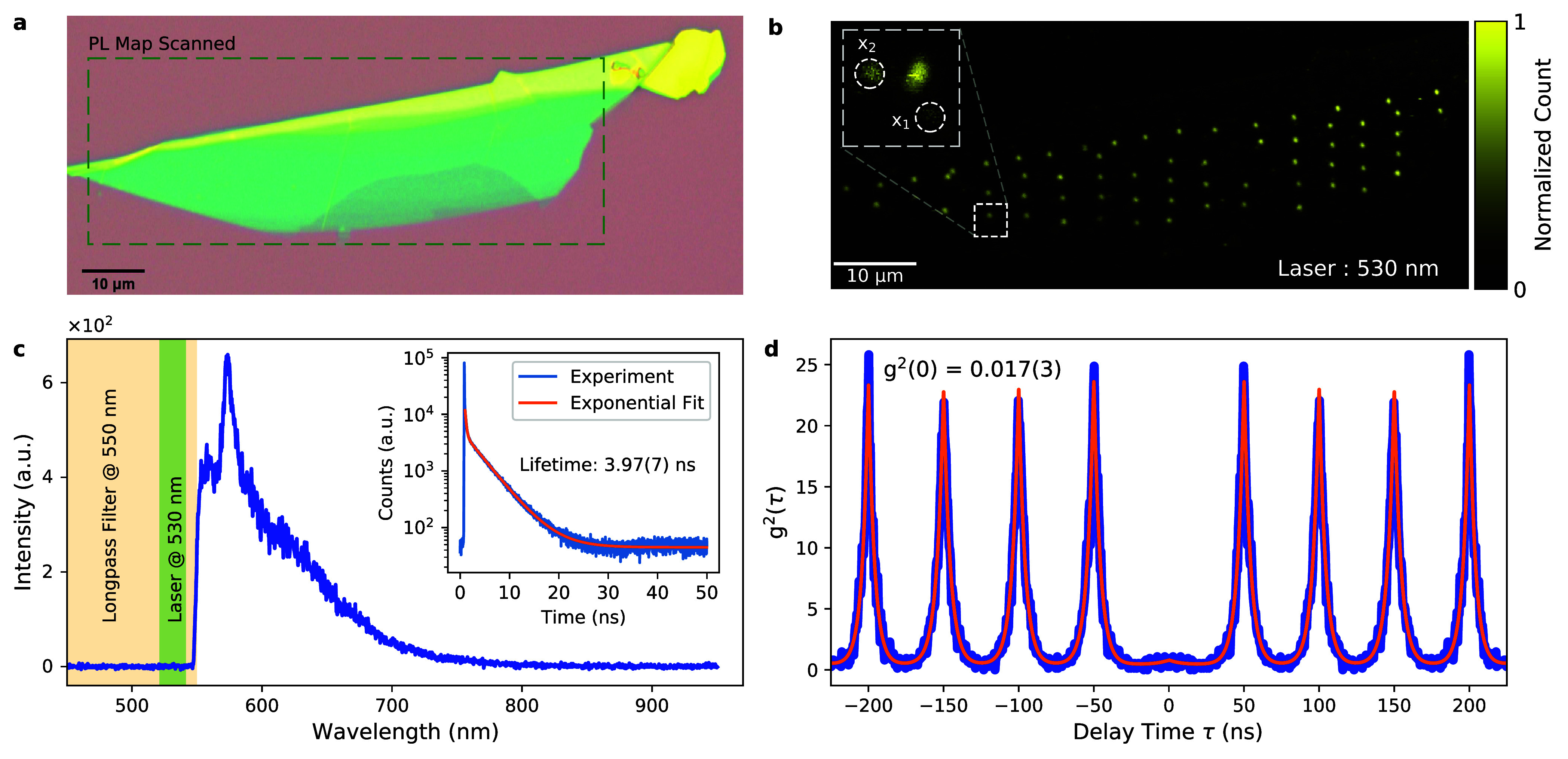
(a) Optical microscope image of the exfoliated hBN flake on a Si/SiO_2_ substrate. (b) PL map of the irradiated array, excited with a 530 nm pulsed laser at a repetition rate of 20 MHz. The inset image is a zoomed-in PL map of one of the irradiated spots, revealing multiple single-emitter spots. (c) Typical spectrum of a single emitter with a peak of the PL at 575 nm, detected with a long-pass filter at 550 nm, that cuts the emission partially. The inset figure shows the typical lifetime decay curve, revealing a lifetime of 3.97(7) ns. (d) The second-order correlation function under pulsed excitation at the position marked “x_1_” in (b) with *g*^2^(0) = 0.017(3) and at “x_2_” with *g*^2^(0) = 0.042(2). The *g*^2^(0) values were extracted from the fitted curve.

A thin hBN flake was mechanically exfoliated from the bulk crystal to a silicon substrate with a 298 nm thick thermal oxide layer (see Methods). Optically active emitters were induced using localized electron beam irradiation with a standard scanning electron microscope (SEM) at a chosen spot in the flake.^21^ The emitters were created over the entire flake, independently of the local flake thickness (Supplementary Information (SI) Section S1.1). Figure 1a shows an optical microscope image of the flake, and Figure 1b shows the resulting photoluminescence (PL) map under pulsed laser excitation with a 530 nm laser (see Methods). The PL map revealed diffraction-limited emission spots that originated from the quantum emitters formed during the irradiation and defect formation process. The inset in Figure 1b shows a zoomed-in PL map of one of the irradiated spots, where more than one emitter is present. This was due to the probabilistic nature of the irradiation process. A typical emission spectrum is shown in Figure 1c with a peak at 575 nm (see SI Section S1.2 for the statistics on the spectra within the array). The typical lifetime of the emitter was around 4 ns (see inset). Due to the long-pass filter that was used, which suppressed the excitation laser, we did not have full access to the spectrum. We still expect that there is not much emission below 550 nm, as excitation with a 470 nm laser in a separate measurement was very inefficient (see SI Section S1.3), implying that there were no available phonon modes in the blue region. The polarization-resolved second-order correlation measurement in Figure 1e proves single-photon emission with *g*^2^(0) = 0.0171(3) for the spot labeled “x_1_” in Figure 1b (and 0.0410(6) for “x_2_”) without any background correction (see SI Section S1.4). The polarization-resolved measurements allowed us to selectively excite the emitter efficiently by matching the laser polarization and, at the same time, suppress any uncorrelated noise sources nearby. This, in turn, led to a better single-photon purity compared to that achieved with measurements with a fixed (but random relative) polarization, as in previous experiments.^21^ All emitters in the array had near-identical photophysical properties (see SI Section S1 and the following sections), which allowed us to study the polarization dynamics.

### Correlation of Emitter Polarization with Crystal Axis

To study the excitation dipole axes of our emitters, we first polarized our laser circularly using a quarter-wave plate. The actual excitation laser polarization was then set using a linear polarizer with a high extinction ratio. The initial circular polarization ensured equal excitation power independent of the current polarization. This combination yielded a more accurate excitation polarization compared to using a simple half-wave plate (see SI Section 2.1). The laser power was monitored using a power meter with a variation below 5%. The polarizer was rotated from 0° to 360° in steps of 10° or 15° by using a motorized mount. A fitting routine of the data allowed us to extract the polarization directions with much higher accuracy than the rotation step size (see SI Section 2.2). We noticed a small beam shift in the PL map during the rotation, likely caused by the optical component not being not plane-parallel. Instead of simply recording the PL count rate using the single-photon avalanche diodes (SPADs), we recorded local PL maps and integrated the intensity of the diffraction-limited spots (see SI Section S2.3). This way, we compensated for the slight beam shift. In the detection path, we used another motorized polarizer to measure the emission dipole axes. Note that this polarizer was present only during the emission dipole measurements and not during the excitation measurements.

For measuring the emission dipoles, the laser polarization was set to have a maximal overlap with the excitation dipole. Once the excitation polarizer was optimized, we recorded the time trace (PL signal over time) while rotating the polarizer with a dwell time of 5 s in the detection path. Afterward, we extracted the integrated PL intensity (see SI Section S1.4). In some cases, we observed multiple polarization axes, which could be due to the presence of multiple emitters within a diffraction-limited spot (see SI Section S1.5). However, we omitted such cases from our analysis and only considered emitters with a clear *g*^2^(0) dip and unique polarization axes.

**Figure 2:**
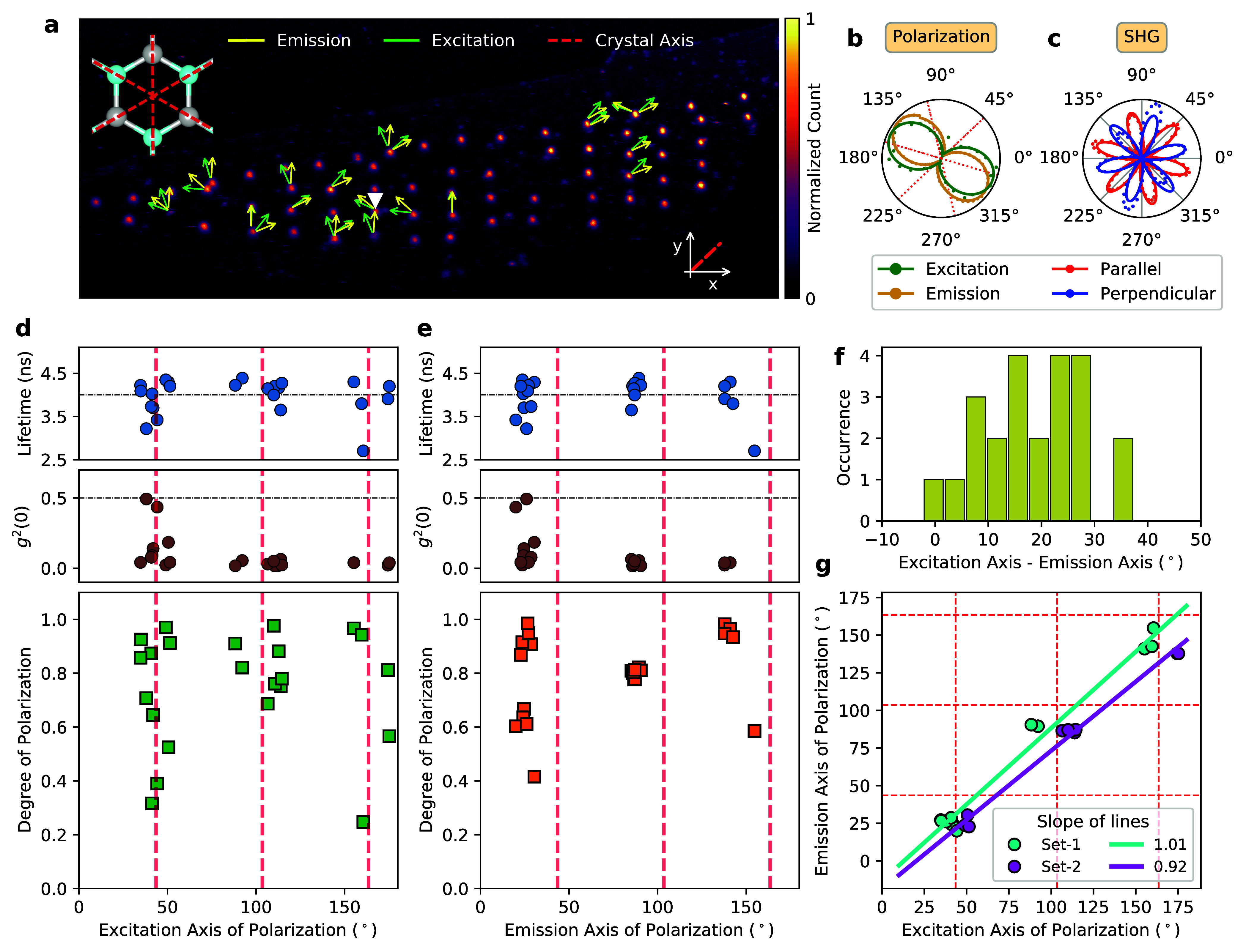
(a) PL map of the entire flake created using a pulsed excitation laser at 530 nm. The emission and excitation axes of the measured emitters are presented with arrows at the measured angle relative to the (random) *x*-axis, as marked in the map. One of the main crystal axes had an angle of 43.52° ± 0.39° with respect to the *x*-axis. (b) A typical polar plot of the emission and excitation axes at the spot marked with a white “▼” in (a). The degree of polarization was extracted from a cosine-squared fit with 98.01% (emission) and 96.67% (excitation). Here, the red and blue grid lines present the crystal axis in order to correlate the emission and excitation axes with respect to the crystal axis. (c) The polarization-resolved SHG measurements revealed the crystallographic axes, as evident by the 6-fold symmetry. These axes are also marked in all subplots. Scatter plots of the measured (d) excitation and (e) emission axes against the degree of polarization of the emitters. All of the emitters presented in the scatter plots have a clear *g*^2^(τ = 0) dip and an average lifetime of around 4 ns, as indicated in the plot. (f) The misalignment between the excitation and emission axes of polarization with a mean value of 18.9(100)°. (g) Emission versus excitation axes showing a linear behavior. The plot shows a clear splitting into two groups identified as “Set-1” and “Set-2”, which both have a slope of nearly 1.

Note that we always specify the polarization axis and not the dipole axis (which was rotated by 90° from the polarization axis). The PL map with green/yellow arrows respectively marking the excitation/emission polarization axes is shown in Figure 2a, as extracted from the fitting routine. Figure 2b shows a typical polar plot of the measured and fitted data from the emitter marked with a white triangle in Figure 2a. We also extracted the error bars from our fitting, which turned out to be smaller than the symbol size and were thus are omitted in Figure 2 (more details can be found in SI Section S3.1). We also observed that the orientation of the excitation/emission axes was independent of the flake thickness (see SI Section S3.2). This distribution of polarization axes was expected due to the specific order of layer stacking in (crystalline) hBN.^33^ The layer orientation or individual local flake thickness had, therefore, no influence on the polarization axes of the defect centers.

The question about how the dipole aligns within the crystal lattice arose. This could be easily probed with polarization-resolved second-harmonic generation (SHG) measurements,^34^ which revealed the 6-fold axis symmetry of the hBN lattice (see Figure 2c). The quadratic pump power dependency verified the second-order process of SHG (see SI Section S4). The crystallographic axes are also indicated in Figure 2a,b with dashed red lines.

We recorded both excitation and emission dipoles for 23 emitters. The scatter plots of the excitation and emission angle (modulo 180°) distribution are shown in panels d and e of Figure 2, respectively. For every emitter, we also measured the g^(2)^(0) value and the excited-state lifetime to verify that we had single emitters, which relax through the same decay channel (see the top part of Figure 2d,e and SI Sections S1.5 and S1.6 for the raw data). We also extracted the degree of linear polarization from our fits and displayed this in the histogram. It is worth noting that with our measurements, we only projected onto the equatorial plane of the Poincaré sphere. A full quantum state tomography would require projecting onto the circular components as well. For simplicity, we refer to the degree of linear polarization simply as the degree of polarization. Many emitters had a high polarization visibility above 80%. It is clear that for the yellow emitters, the excitation and emission axes bunch around certain angles in relation to the crystal axis with an uncertainty range of 8° for excitation and 4° for emission. The misalignment between excitation and emission was, on average, 18.9(100)°, as shown in Figure 2f. This large uncertainty made it difficult to assign a specific defect complex (which we, therefore, did not attempt). In general, this could be due to multiple involved transitions or local modifications in the crystal environment and needs further investigation.

We also observed a splitting between the excitation and emission axes of polarization, and this became even clearer when the emission axis was plotted versus the excitation axis in Figure 2g, where six groups could be distinguished. If the excitation/emission polarization co-aligns with a crystal axis or is exactly in the middle of the crystal axis, a 3-fold symmetry results (i.e., three groups). If there is an angle between the polarization and crystal axes, these three groups split into six symmetrically around the crystal axis. In our case, however, the centers of the groups were not separated by 60° (for emission), and the mean distances from the crystal axis ranged from roughly 3° to 10° (SI Section S5). Moreover, the splitting in the emission polarization was less prominent and not symmetric around a crystal axis (unlike that in the excitation polarization). This symmetry breaking could be due to localized strain in the crystal lattice that was induced during the localized electron irradiation process or other local modifications of the crystal environment. It is important to note that we observed some anisotropy in our SHG measurement in Figure 2c, which is related to residual strain in the crystal lattice.^35^ However, this is the global strain that is typically induced during the exfoliation process. The local strain, in particular around the irradiated spots, could be considerably higher and was not resolvable with our SHG setup. Such strain could also lead to a change in the polarization axis,^27^ and to further investigate it, we modeled this qualitatively using DFT.

### Temporal Polarization Dynamics

**Figure 3:**
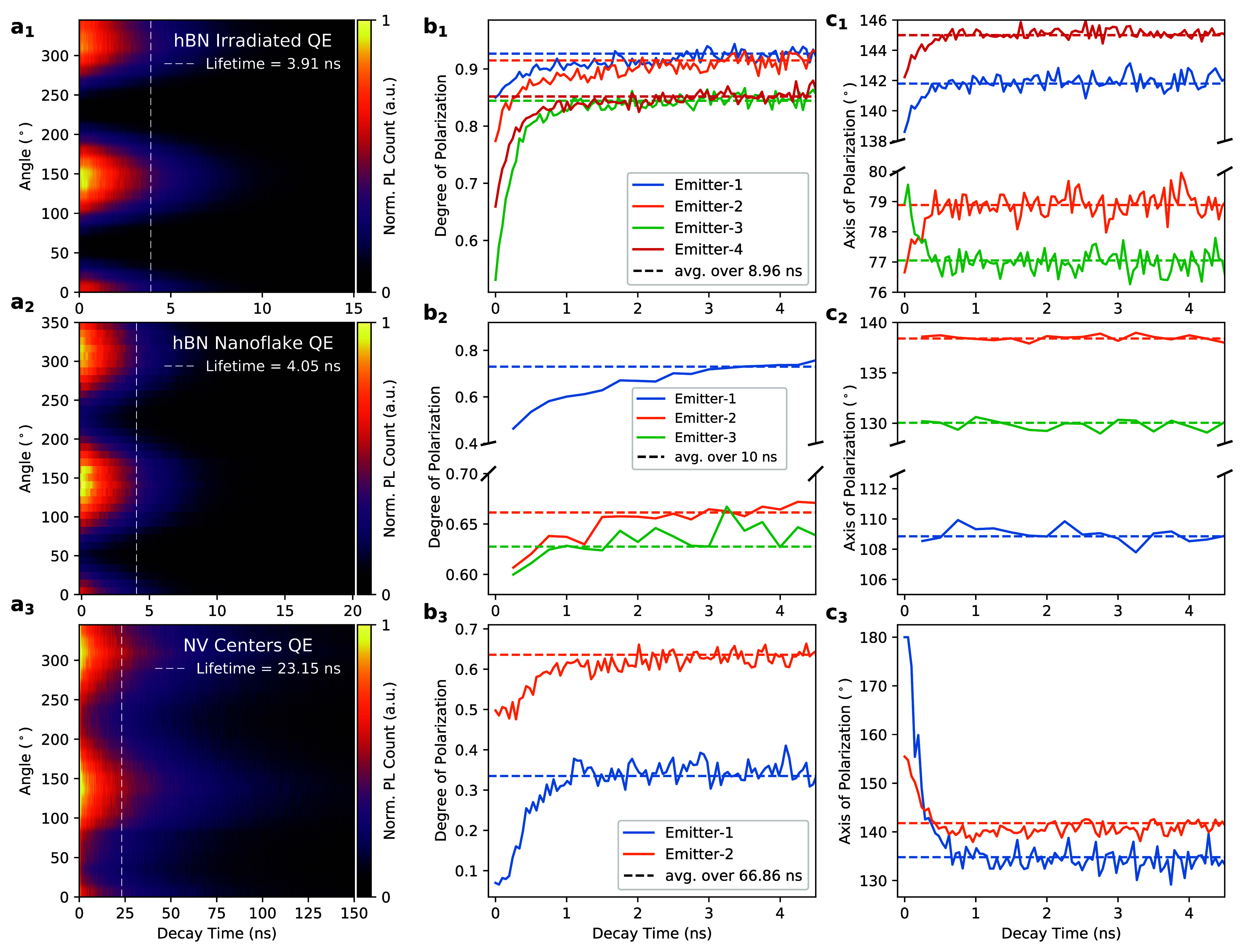
(a) The PL intensity with respect to time after the excitation laser pulse as a function of the polarizer rotation angle measured for (a_1_) hBN irradiated QEs, (a_2_) hBN nanoflake QEs (with *g*^2^(τ = 0) values well below 0.5), and (a_3_) NV center ensembles in diamond (with *g*^2^(τ = 0) above 0.5). The dashed lines represent the extracted lifetime of the emitter. (b) The variation of the linear degree of polarization and (c) the axis of polarization measured with respect to time slices (time spent in the excited state) for different emitters. The dashed lines indicate the time-averaged visibility and polarization axis obtained by integrating over an extended time period (i.e., the results observed in Figure 2). For the irradiated hBN emitters and NV centers, measurements were obtained with a pulsed excitation of 530 nm at 20 and 10 MHz repetition rates, respectively. Measurements for the hBN nanoflake emitters were obtained under 483 nm pulsed excitation at a 10 MHz repetition rate.

We now turn our focus to the investigation of temporal polarization dynamics of the hBN (and, in general, of solid-state) quantum emitters. This study of the temporal emission polarization dynamics was performed by recording the decay curve as a function of the rotation angle of the polarizer in the emission path. The algorithm that was used to extract the relevant time-resolved polarization dynamics is described in SI Section S6. As we were also interested in whether any observed effect was generic or only a sample-specific artifact, we also repeated this measurement for hBN nanoflakes and NV centers in nanodiamonds. The general optical characterization of these samples is shown in SI Sections S7 and S8. Each decay measurement that corresponded to a different polarizer angle was then combined to obtain a polarization-resolved decay map. Figure 3a shows exemplary polarization-resolved decay maps from three different emitter types. Each map was then divided into time bins, and a generic cosine-squared function was fitted into each individual time bin to extract the linear polarization visibility and the polarization axis (panels b and c of Figure 3, respectively) as a function of the time that the charge carrier had spent in the excited state. In order to account for the instrument response function (<70 ps full width at half-maximum (fwhm)), the initial 120 ps of the data was omitted from the analysis.

Interestingly, for all emitters investigated including the NV centers (we used ensembles with 1–4 NV centers), we observed a strong increase in the linear polarization visibility (see Figure 3b) during the first 1–3 ns. Accompanied by the visibility change, most of the emitters also showed rotation in the polarization axis with respect to the decay time, while the polarization of others remained stable during this time scale. These effects could be caused by photoinduced modifications of the local charge environment around the emitter. In other words, the laser pulse excited other optically inactive nearby emitters or charge states, which resulted in an induced electric field around the emitter.^36^ Such electric field fluctuations could have temporarily shifted the dipole axis of the emitters, resulting in a decrease in the observed polarization visibility. Alternatively, the pulsed excitation laser that was used in all our measurements carried a very high peak laser power in every pulse. This could have also induced local strain or structural fluctuations in the crystal for a short period of time, which then could have led to temporal dynamics of the dipole polarization. Moreover, the pump laser illumination can also modify the electron occupation distribution around the defects independent of local charges in the surrounding environment. If these were true mechanisms, then this should be laser-power-dependent. Our power-resolved measurements (see SI Section S9) did not indicate this; however, we are not necessarily ruling this out, as the effect could already be saturated at all studied laser powers. In this case, when these undesired excitations or photoinduced modifications or strain relax on fast time scales, the visibility and polarization axis reach their steady states. Importantly, the time scale of the relaxation was much longer than the laser pulse length (<70 ps fwhm) and varied from sample to sample. This could indicate different local charge distributions in the crystal environment or modifications in the crystal environment due to laser illumination.

We would also like to remark on some of the discrepancies between the polarization axis results of different samples. Observations showed that both irradiated hBN emitters and NV centers (panels c_1_ and c_3_ of Figure 3, respectively) showed a clear change in the polarization axis, whereas emitters in the hBN nanoflakes (Figure 3c_2_) did not show such a change. If these changes were indeed affected by the local environment of the emitter, then we would expect sample preparation to play a crucial role in these different observations. The hBN nanoflakes were less clean in comparison to the irradiated hBN quantum emitters and the NV centers in diamond in terms of local strains, charge states, or even nearby emitters. If the orientation of the dipole could be affected by its local environment in a given time, we would expect such interactions to statistically average out each other in terms of dipole orientation. This would explain both the visibility change and the orientation stability of the hBN nanoflakes. In the case of the irradiated hBN samples, we would expect the local environment to be much cleaner and more ordered in comparison to the nanoflakes. In the absence of such heavily random interactions, one might expect the crystal axes to play a major role as a static force on the dipole orientation. Again, in a given time, the emitter might fluctuate its orientation in a preferred direction, but such fluctuations in the orientation would result in a decrease in the observation of the visibility and orientation. The same discussion can also explain rotational changes in the orientation (Figure 3c_1_, green line) since the rotational difference between an emitter and the (closest) crystal axis can be either +*X* or −*X* degrees.

It is worth mentioning that our experiments recorded the emission dipole orientation with respect to a fixed excitation dipole. The effect of the excitation laser polarization dependence on the temporal dynamics is still unknown. A similar mechanism of temporal dynamics of polarization could exist for the excitation dipole, but it is challenging to distinguish from the emission temporal polarization effect. Finally, we draw attention to the results of the NV centers, which are a completely different type of quantum emitter that surprisingly showed the same behaviors as the hBN emitters. The NV center is a very well-studied system, yet we are not aware of any such effect being reported. This raises the question of whether these observations are generic to other emitter types in other materials, such as quantum dots,^37^ quantum emitters in two-dimensional (2D) transition metal dichalcogenides (TMDs),^38^ and three-dimensional (3D) crystals such as diamond and silicon carbide.

### Polarization Dynamics with Density Functional Theory

We now turn to theoretically modeling the observed effects with DFT. This section answers the following questions: (i) why are the excitation/emission dipoles misaligned? (ii) How do the dipoles specifically align with respect to the crystal axes? (iii) Can strain cause symmetry breaking? Lastly, (iv) can electric excess charges/defects cause temporal variations? We address these questions using spin-polarized density functional theory. Our DFT calculations used the HSE06 functional (see Methods), which provides reasonable accuracy for calculating the electronic band structures of hBN quantum emitters, as verified by experiments, compared to functionals from the generalized gradient approximation.^39^ We studied the most likely candidates, e.g., intrinsic defects and complexes involving oxygen and carbon impurities with neutral or ±1 charge states. Carbon complexes could form during the SEM irradiation process, consistent with previous DFT calculations yielding 2 eV quantum emitters in hBN.^23,40,41^

**Figure 4:**
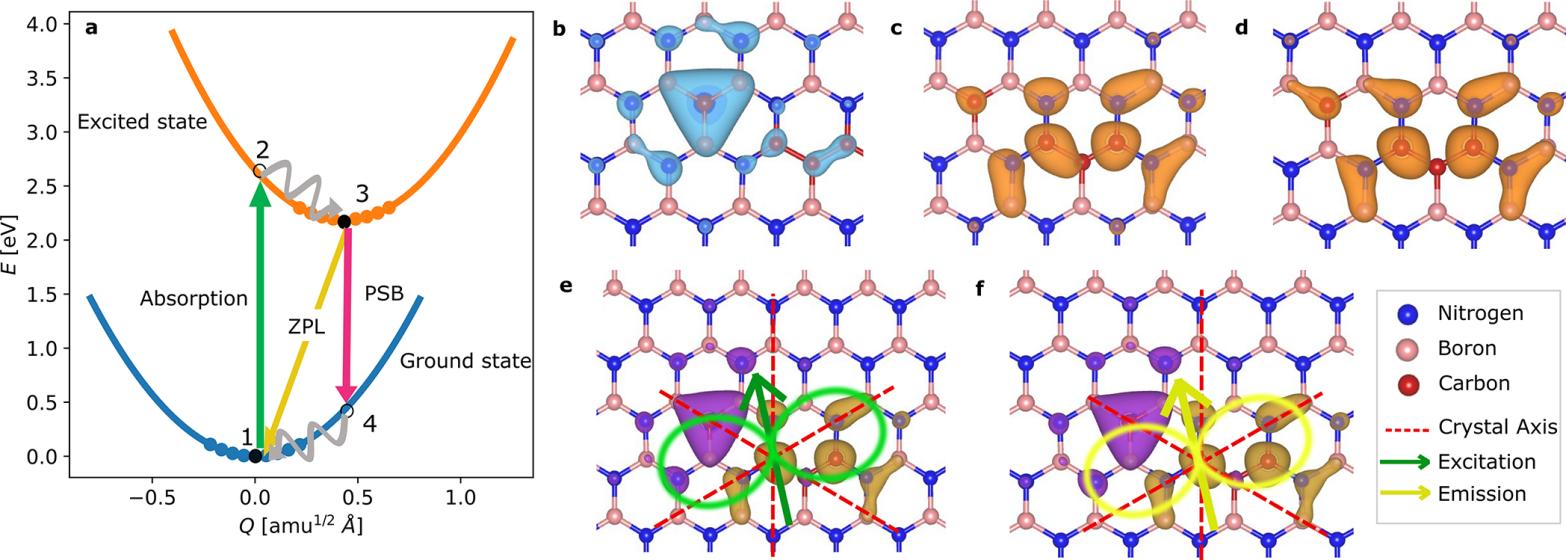
(a) Potential energy surface of a neutral-charged C_2_C_2_ defect without strain, representing the complete excitation and emission process, consisting of the absorption (green line), the zero-phonon line (ZPL, yellow line, here 573 nm), and the phonon sideband (PSB, magenta line). (b–d) The probability density |ψ|^2^ of electron occupations in the ground state at point 1 and in excited states at points 2 and 3, respectively. (e) The charge difference between points 1 and 2 is shown by the isosurfaces, where the green arrow indicates the excitation dipole axis with light radiation in green shade. The excitation axis makes a 11.1° angle relative to the crystal axis (red dashed line). (f) The charge difference between points 1 and 3 with the yellow arrow indicating the emission dipole axis with light radiation in yellow shade. The emission axis makes a 12.1° angle relative to the crystal axis.

The electronic transition of a defect, in theory, can be described by the Huang–Rhys model, as shown in Figure 4a, where the ground and excited states, depicted by the blue and orange curves, respectively, are responsible for the transition. Each state consists of vibrational modes, illustrated by the dots on the respective curve. In principle, a transition between any pair of ground and excited states is possible, resulting in the absorption and emission spectrum. Both consist of the zero-phonon line (ZPL) and a phonon sideband (PSB). We note that the transition dipole moment for the absorption can depend on the specific phonon mode (i.e., it is affected by the excitation laser wavelength relative to the ZPL).^42^ The excitation is the transition from points 1 to 2 in Figure 4a. The system will relax to point 3 on ultrafast time scales (typically on the order of a few ps) through phonon scattering. From point 3, the emission can take place either directly to the ground state (point 1) or via another phonon mode (point 4). We have calculated both transition dipole moments for the ZPL (3–1 transition) and a (randomly chosen) phonon mode in the PSB (3–4 transition) and found only a negligible difference in the relative angles (below 1°, see SI Section S10). In the experiment, one would see the average (i.e., the averaged dipole over ZPL and PSB, which has a lower polarization visibility in case of a misalignment between both transition dipole moments). Hence, we restrict the following analysis to the ZPL transition (points 3 to 1).

As these particular points originate from different electronic states and ionic relaxation configurations, their wave functions can be distinct. This is also consistent with previous DFT calculations.^43^Figure 4b–d shows the probability densities (|ψ|^2^) of the electron occupation of a defect (C_B_C_N_C_B_C_N_) corresponding to points 1 to 3, respectively. The differences between points 1 and 2 as well as points 3 and 1 are shown in panels e and f of Figure 4, respectively. As the transition dipole moment is proportional to ⟨ψ_f_|**p**|ψ_i_⟩ (see eq 1 in Methods), where i and f denote the initial and final states of the transition, respectively, distinct wave functions can lead to different dipole moments and, therefore, to an angle between the excitation and emission polarization. Hence, this answers question (i). We are nevertheless not ruling out that, even in our case, the misaligned dipoles can be caused by additional intermediate states, as has been reported before.^42^

To identify the type of defect matching the experimental observations, we calculated the properties of 126 native, carbon-, and oxygen-based defects with different charge states (see SI Section S10 and the SI data set) and applied the following criteria to select the most promising defect candidates: ZPL range, orientation of the absorption polarization axis relative to the crystal axis (in the range of 3.9° to 11.6°), and the linear in-plane polarization visibility. We restricted these criteria to the absorption dipole, as our data were consistent with an excitation dipole having a finite angle relative to the crystal axis. The interpretation of the emission data is discussed later in this work. The choices of oxygen and carbon for the impurities were based on the fact that they have been suspected to be responsible for the 2 eV emission^44,45^ and because of their natural chemical stability. Among the 126 studied defects, only 22 satisfied the range of the excitation polarization; however, most of these could be additionally ruled out due to the polarization visibility. In particular, many of the charged defects exhibited strong out-of-plane contributions. We found that the dipoles of charged-state defects were impacted by free excessive positive/negative charges and out-of-plane structural deformation, leading them to align perpendicular to the crystal plane. This finding also confirmed that the dipoles depend on the charge distribution and structural deformation. The remaining candidates could further be narrowed down when the ZPL was taken into account (see SI Section S10). This essentially eliminated all defects except for the C_B_C_N_C_B_C_N_ defect complex. We hereafter abbreviate this complex as C_2_C_2_. Due to having four carbon defects involved, it can exist naturally in several configurations (see SI Section S10.4), which we denote with numbers, i.e., C_2_C_2_-*n*. The most likely configurations were the neutral-charged C_2_C_2_-3 (which is shown in Figure 4) and the neutral-charged C_2_C_2_-5 with ZPLs at 573 and 562 nm, respectively. With our current theoretical and experimental uncertainties, we cannot distinguish these cases definitely, and in principle, it would even be possible to have a mixture of both cases present in our sample. The atomic structures of these configurations are shown in SI Section S10. Their excitation (emission) polarization axes aligned 11.1° (12.1°) and 12.3° (13.7°) relative to the nearest crystal axis, as marked by the arrows in Figure 4e,f. This small difference between the excitation and emission polarization axes is not consistent with the experimentally observed difference, which could be due to multiple factors causing the distortion of the charge distribution in the experiments, such as an inhomogeneous distribution of strain in the flake or localized charge in the lattice due to the irradiation process. It is important to note that these structures undergo essentially only in-plane deformations during relaxation, making them inherit pure in-plane dipoles. Of course, the polarization can, in principle, be out-of-plane; nonetheless, our calculations indicate in-plane polarization. We therefore propose the C_2_C_2_ defect to be responsible for our emission, even though it exhibits notable variations in dipole angles compared to the experimental observations. However, when considering the overall properties (and also the ZPL, polarization visibility, etc.), the C_2_C_2_ defect emerged as the most favorable emitter compared to all of the other studied defects (see SI Section S10). This also provides the answer to question (ii).

What our model so far could not explain is the symmetry breaking, i.e., the center of the groups in the emission axis not being centered around the crystal axes and also not being spaced by 60° as well as the large distribution of, for example, the angle difference (excitation – emission). We speculate that this could be caused by strain. One has to distinguish two cases here: global strain and local strain. We know the former is not significant, as otherwise, the SHG pattern would be skewed. Nevertheless, there could be a significant amount of local strain around the irradiated spots. We could model whether this was possible qualitatively using DFT as well. We (theoretically) applied biaxial strain in the range of ±1% to the lattice and monitored the dipole orientations. For vacancy based defects, the shifts could amount to >4°, while for the C_2_C_2_-3 defect, for example, this remained below 0.5° in the investigated strain range (see SI Section S10). Moreover, we also observed that the excitation and emission dipoles were affected differently by local strain, which could, in part, account for the symmetry breaking and, hence, the unequal shifts from the crystal axis. We can therefore answer question (iii) only qualitatively in part and attribute either large local strain around the irradiated spots or other (so far unknown) local modifications in the crystal environment as the cause of the symmetry breaking. A quantitative description of this is beyond the scope of this work and will be carried out by us in a future study.

Finally, this leaves the question of polarization (question (iv)) to be answered. To model the temporal variations observed in Figure 3, we applied an external electric field (up to 0.7 V/Å) to mimic the redistribution of the local charge environment around the defects. The results (see SI Section S10) indicated that for all defects, the in-plane dipoles turned out to have a high out-of-plane contribution with high intensity of an out-of-plane electric field. We only considered the limit of weak electric fields that only have a minor impact on the photophysical properties (i.e., adiabatic changes only and no jumps). In this limit, the reduction in visibility can be substantial (>20%), along with a polarization axis of rotation > 5°. This confirms (again qualitatively) that the dipoles are sensitive to the local charge distribution, and this could explain why the polarization visibility increased when reaching the steady state, as depicted in Figure 3c.

## Conclusion

The present work demonstrates an in-depth study of the polarization dynamics of identical yellow single-photon emitters in hBN, which were fabricated using a standard electron beam microscope. Our findings indicate a correlation between the excitation and emission axes and the crystallographic hBN crystal axis. While the excitation polarization bunches around the crystal axes, we found that the emission polarization bunches between the crystal axes. As the latter groups are not separated by 60°, we suggest that local strain could cause symmetry breaking. The correlation of the crystal axes with the dipole polarizations of the quantum emitters can, in principle, be used to identify the emitter in question when compared with predictions calculated with DFT. The direct identification of hBN quantum emitters has been shown to be technically difficult in the past. While in this work we had a large variation in the observed polarization angles to undoubtedly identify the emitter, we were able to narrow down potential defect candidates. When this was done together with other photophysical properties such as emission spectrum, this led to a convincing case for the proposed atomic structure to be responsible for the 2 eV quantum emitter in hBN.^46^ This could also provide an approach to address the atomic structures of fluorescent defects in other materials systems, such as TMDs,^38,47,48^ silicon carbide,^49^ and silicon.^50^

We also investigated the temporal dynamics of the polarization of single photons generated from defects in irradiated and nanoflake hBN as well as the negatively charged nitrogen vacancy center in diamond. A higher degree of emission polarization was observed for the carriers that stayed in the excited state longer. We speculate that this effect could be due to the local electric field induced by excess charges that are excited with the laser pulse. This effect can also be explained in terms of photoinduced strain or modifications in the local charge distribution under pulsed excitation. To complement our experimental observations, we provided DFT calculations. Nevertheless, spin Hamiltonian simulations are further required for studying the polarization dynamics. We believe that the observed temporal change of polarization in various solid-state quantum emitter systems is critical for reaching the ideal performance of these emitters for several applications, such as generating Fourier-transform limited photons^51,52^ or achieving a lower quantum bit error rate in quantum key distribution systems.^31,53^ It might even be an important step toward achieving indistinguishable single photons from a room-temperature solid-state quantum light source when coupled with resonant structures.^54^

## Methods

### Emitter Fabrication

A multilayer hBN flake was exfoliated from a bulk crystal (HQ Graphene) using the scotch tape method onto a viscoelastic polymer sheet (polydimethylsiloxane) purchased from Gel-Pak (WF-40-X4). The exfoliated flakes were examined under a bright-field optical microscope to identify a suitable thin flake based on optical contrast. Afterward, the flake was transferred onto a grid-patterned Si/SiO_2_ substrate with a 298 nm thermal oxide layer. This grid was fabricated by using electron beam lithography and a metal lift-off process, which allowed us to easily navigate on the substrate.

The nanoflake emitters were obtained in solution with a concentration of 5.5 mg/L from the Graphene Supermarket. The number of atomic layers per flake varied between one and five, with a typical flake diameter ranging from 50 to 200 nm. Approximately 10 μL of the solution was drop-cast onto a Si/SiO_2_ substrate with a 300 nm oxide layer and dried under ambient conditions. No further post-processing, such as high-temperature annealing, was carried out.

The nanodiamonds were prepared by drop-casting a commercially available nanodiamond solution (Adámas Nanotechnologies, 40 nm Carboxylated Red FND 1–4 NV per particle) on standard glass substrates, which were then stored at ambient conditions overnight to dry.

### Emitter Irradiation

The emitter array was produced by using a scanning electron microscope (Helios NanoLab G3). The electron beam was accelerated at 3 kV with an electron current of 25 pA. These settings were used for beam alignment, imaging, and actual irradiation. The imaging of the flake was carried out with an electron fluence of 1.4 × 10^13^ cm^–2^. To fabricate the emitters, a high electron flux was pointed for a dwell time of 10 s (fluence of 7.7 × 10^17^ cm^–2^) onto pre-defined spots on a suitable flake.

### Optical Characterization

The optical investigation of the hBN emitter array and the NV samples was carried out using a commercial fluorescence lifetime imaging microscope (PicoQuant MicroTime 200) with a 530 nm pulsed laser at a 20 MHz repetition rate and a pulse length below 80 ps (fwhm). For the NV samples, we reduced the repetition rate to 5 MHz (to account for their higher excited-state lifetime). Unless stated otherwise, the excitation power for all measurements was around 50 μW (peak power was ≥10 mW). For the PL mapping, which was done using a scanning stage, a dwell time of 5 ms/pixel was used. The laser was circularly polarized with a quarter-wave plate and then linearly polarized using a nanoparticle film polarizer on a motorized mount. The PL signal was collected using a 100× dry immersion objective with a high numerical aperture (NA) of 0.9 and a working distance of 0.3 mm. In the detection path, we inserted a long-pass filter to suppress the excitation laser and another motorized nanoparticle film polarizer. The photons were detected by two single-photon avalanche diodes from Micro Photon Devices or a high-resolution spectrometer. The assembly of the SPADs in both arms of a 50:50 beam splitter enabled us to measure the second-order correlation function. The data analysis of the correlation function as well as the lifetime measurements was performed with the built-in software (which also took the instrument response function into account by convoluting the initial fit function with the measured instrument response function of <70 ps fwhm and then using the resulting function to fit the data). The spectral data were obtained with an acquisition time of 1 min/emitter.

The optical properties of the hBN nanoflake emitters were studied by using a custom-built confocal microscope setup. The setup comprised various pulsed lasers (Advanced Laser Diode Systems, Pilas) with wavelengths of 405, 483, and 637 nm and pulse lengths below 50 ps, an objective with an NA of 0.75 and 50× magnification, a spectrometer with a resolution of 0.03 nm (Andor, Shamrock 750) together with a charge-coupled device (CCD) camera (Andor, Newton), and four SPADs (ID Quantique, 2× ID120, and 2× ID100), which were located at the detection ports. The excitation power used in all measurements was around 100 μW unless stated otherwise, which was below the saturation power of the emitters. A combination of long-pass and notch filters was used at the detection port to filter out the excitation laser, while various bandpass filters were employed to selectively filter out the PL emission from different emitters. Time-correlated single-photon counting was performed using a time-tagger module (Roithner LaserTechnik TTM8000) with a resolution of 41 ps to record the event times. To prevent intensity variations dependent on acquisition, a fixed acquisition time of 1 min was used for each polarizer angle in the temporal polarization measurements.

During all temporal polarization measurements, the signals were filtered by either spectral filters or a spectrometer to exclude the excitation laser that was reflected from the sample surface. Additionally, we also monitored the antibunching measurements for the emitters.

### SHG Characterization

A pulsed Ti:sapphire laser (Coherent Verdi & Chameleon) was used as the pump source for the SHG measurements. The laser wavelength was set to 800 nm, and the pulse duration was 200 fs (estimated at the sample position) at a repetition rate of 76 MHz. The power was controlled by a half-wave plate (HWP) and a polarizing beam splitter (PBS). The pump laser polarization was controlled by a motorized HWP and was coupled to the sample with a beam splitter (BS) and a 50×, 0.55 NA objective (Zeiss LD EC Epiplan-Neofluar). The reflection at 800 nm (pump) and the transmission at 400 nm (second-harmonic) of this BS were similar for both polarization components. The sample was mounted on an *XY*-motorized stage for position control, and the objective was on a *Z*-axis motorized stage to control the focus. The generated second-harmonic light was coupled from the sample in reflection using the same objective and separated from the pump by the BS. A motor-controlled polarizer enabled polarization analysis of the SHG process, and a spectral filter (BG39) was used to remove excess pump light before detection with a thermoelectrically cooled CCD (Andor Zyla 4.2P). The average power of the Ti:sapphire laser was set to 20 mW before the objective, which was sufficient for observing the SHG signal and low enough to not damage the hBN flake. The polarization scans involved rotating the HWP and polarizer in either a parallel configuration  or a perpendicular configuration . At each polarization setting, the CCD intensity was integrated for 10 s, and finally, a background subtraction with the pump laser turned off was used to improve the contrast of the data.

### DFT Calculations

All spin-polarized DFT calculations were performed using the Vienna Ab initio Simulation Package (VASP) with a plane-wave basis set^55,56^ and the projector augmented wave (PAW) as the pseudopotentials.^57,58^ The sizes of the vacuum layer and supercell were optimized until the hBN band structure remained unchanged, which yielded a 15 Å vacuum layer and a 7 × 7 × 1 supercell size containing 98 atoms. The HSE06 functional was employed for all calculations, as it is known to yield more reliable results with the experiment than the generalized gradient approximation.^39,59^ The single Γ-point calculation was implemented to relax the structures with only internal coordinates allowed until the force was lower than 0.01 eV/Å. All geometry relaxations were performed with an energy cutoff at 500 eV and the total energy convergence with an accuracy of 10^–4^ eV. For the excited-state calculations, we used the ΔSCF method to constrain the electron occupation in the excited-state configuration. The transition dipole moment (TDM)^43^ is expressed by
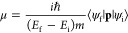
1where *E*_i_ and *E*_f_ are the eigenvalues of the initial and final orbitals, respectively, *m* is the mass of an electron, and **p** is the momentum operator. All other computational details can be found in SI Section S10. Note that because the excitation/emission polarization axes are perpendicular to the dipole axes, we projected and rotated the calculated dipole axes to be consistent with the experiments. To extract the wave function, the PyVaspwfc Python code^60^ and the modified version^43^ were implemented. Finally, we applied an out-of-plane external electric field along with the dipole correction to prevent the error from the periodic conditions for an electric field simulation. To investigate whether strain changed the dipole orientation, biaxial strain was applied. Note that for both the electric field and strain calculations, we set the force to 0.02 eV/Å to reduce computational time.

## Data Availability

All data from this work is available from the authors upon reasonable request. The complete dataset for defect candidates, as determined through DFT, can be accessed through the provided link: https://doi.org/10.5281/zenodo.10288562.

## Supporting Information Available

General photon-physical properties of the yellow emitters; polarization dynamics data acquisition and data analysis; second-harmonic generation measurement; misalignment between excitation, emission, and crystal axes; temporal polarization dynamics; results for the hBN nanoflake quantum emitters; results for the NV centers in diamond; power-dependent temporal dynamics of polarization; and DFT calculation details (PDF)
Complete data set for the properties of all defect candidates, as determined through DFT calculations (XLSX)
