# Supplementary material for: Polarization Dynamics of Solid-State Quantum Emitters
Source: ACS Nano. 2024 Feb 9;18(7):5270–81. doi: 10.1021/acsnano.3c08940 (PMC10883057; doi:10.1021/acsnano.3c08940)
Supplement: Supplementary file 1 — nn3c08940_si_001.pdf [file nn3c08940_si_001.pdf]

Supplementary Information: Polarization dynamics of solid-state quantum emitters

Anand Kumar[1,2,†], Çağlar Samaner[3,†], Chanaprom Cholsuk[1,2], Tjorben Matthes[1,2], Serkan Paçal[3], Yağız Oyun[4], Ashkan Zand[1,2], Robert J. Chapman[5], Grégoire Saerens[5], Rachel Grange[5], Sujin Suwanna[6], Serkan Ateş[3,*], and Tobias Vogl[1,2,*]

[1]Department of Computer Engineering, School of Computation, Information and Technology, Technical University of Munich, 80333 Munich, Germany
[2]Abbe Center of Photonics, Institute of Applied Physics, Friedrich Schiller University Jena, 07745 Jena, Germany
[3]Department of Physics, İzmir Institute of Technology, 35430 İzmir, Turkey
[4]Department of Photonics, İzmir Institute of Technology, 35430 İzmir, Turkey
[5]Optical Nanomaterial Group, Institute for Quantum Electronics, Department of Physics, ETH Zurich, 8093 Zurich, Switzerland
[6]Optical and Quantum Physics Laboratory, Department of Physics, Faculty of Science, Mahidol University, 10400 Bangkok, Thailand
[†]These authors contributed equally.
[*]serkanates@iyte.edu.tr and tobias.vogl@tum.de

Contents

S1 General photon-physical properties of the yellow emitters **3**
S1.1 Correlation of emitter yield with flake thickness and crystal edges 3
S1.2 ZPL distribution 4
S1.3 Emission spectra under 470 nm laser excitation 6
S1.4 Polarization-resolved second-order correlation measurements 8
S1.5 $g^{(2)}$ distribution 8
S1.6 Lifetime distribution 10

S2 Polarization dynamics data acquisition **11**
S2.1 Creation of circular laser polarization 11
S2.2 Fitting Function 11
S2.3 Excitation polarization 12
S2.4 Emission polarization 14

S3 Polarization dynamics data analysis **14**
S3.1 Excitation and emission polar plots of the emitters 14
S3.2 Dipole correlation with flake thickness 15

S4 Second-harmonic generation measurement **16**

S5 Misalignment between excitation, emission axis and crystal axis **17**

S6 Temporal polarization dynamics **18**
S6.1 Sample-1: irradiated hBN emitters 21
S6.2 Sample-2: nanoflake hBN emitters 22
S6.3 Sample-3: NV centers in diamond 23

S7 hBN nanoflake quantum emitters 23

S8 NV centers in diamond 27

S9 Power-dependent temporal dynamics of polarization 28

S10 DFT calculations 29

S10.1 Dipole calculations . . . 29
S10.2 Dipoles of yellow emitter's candidates . . . 30
S10.3 Charged-state defects . . . 32
S10.4 C_2C_2 configurations . . . 34
S10.5 Defect formation energy . . . 35
S10.6 Effects of an electric field on dipole orientation . . . 36
S10.7 Effect of strain on dipole orientation . . . 37
S10.8 Effect of strain on ZPL . . . 38

S1 General photon-physical properties of the yellow emitters

The yellow single photon emitters are fabricated using localized electron beam irradiation using a standard scanning electron microscope (SEM). The general optical characterization of the fabricated yellow emitters is carried out using 530 nm pulsed excitation laser. The more detailed analysis and fabrication methodology is also summarized in Ref. [1].

S1.1 Correlation of emitter yield with flake thickness and crystal edges

We fabricated the emitter array using localized electron beam irradiation using a standard SEM machine which allows us to irradiate at a chosen spot in the flake. In order to understand the generation of the emitter at the irradiated spot, we overlayed the SEM and PL map of the flake as shown in Fig. S1. It is clear that irradiation spots correspond to the bright emission in the PL map. We also overlayed the thickness map of the flake measured by a phase shift interferometer (PSI) with a PL map to further understand the correlation of emitter occurrence with the crystal edges and thickness. Fig. S2 shows that the occurrence of emitters are independent of the crystal thickness and edges in the flake.

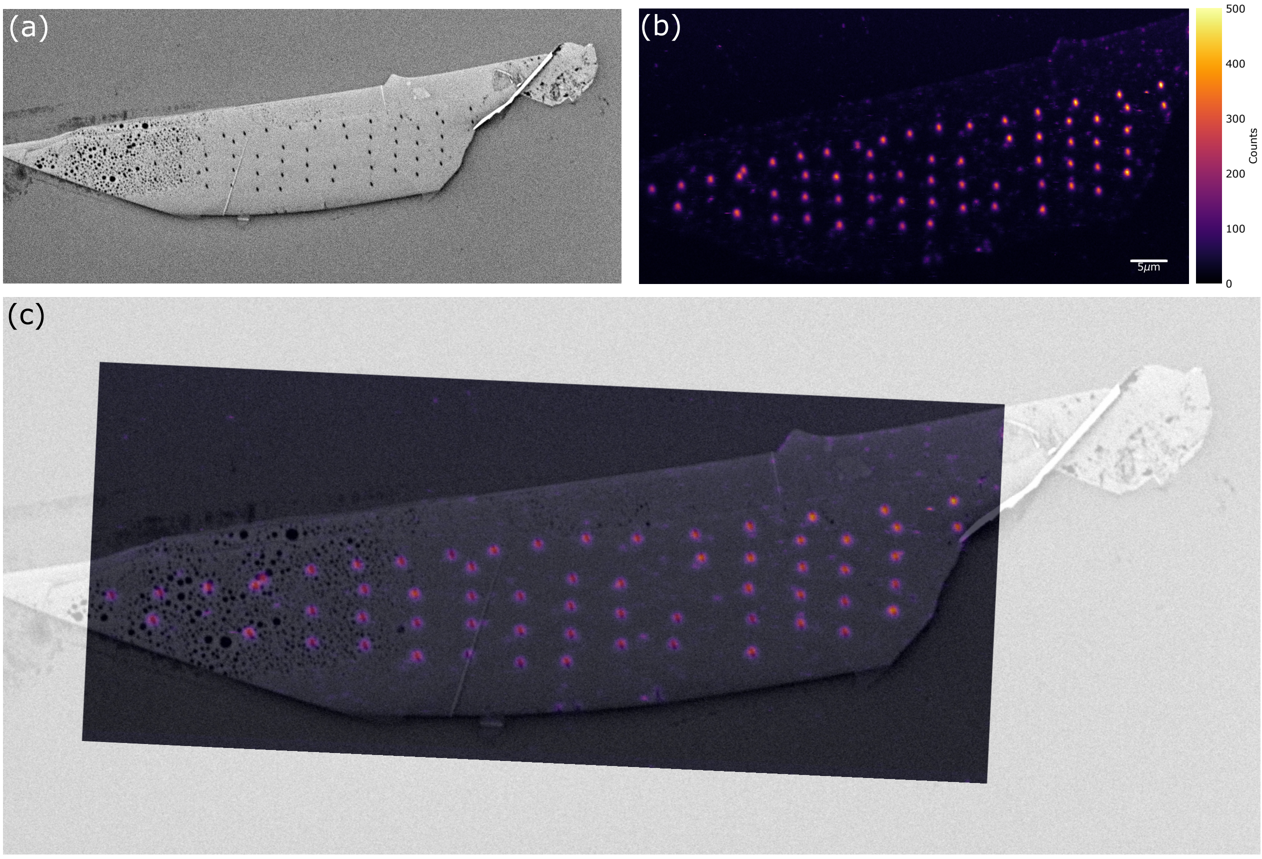

Figure S1: (a) A SEM image of the flake after irradiation. (b) A PL map of the flake is recorded using 50 nm pulsed excitation laser. (c) A overlapped PL and SEM image of the flake revealing bright emission from the irradiated spot. Here the SEM image is set to 30 % transparency.

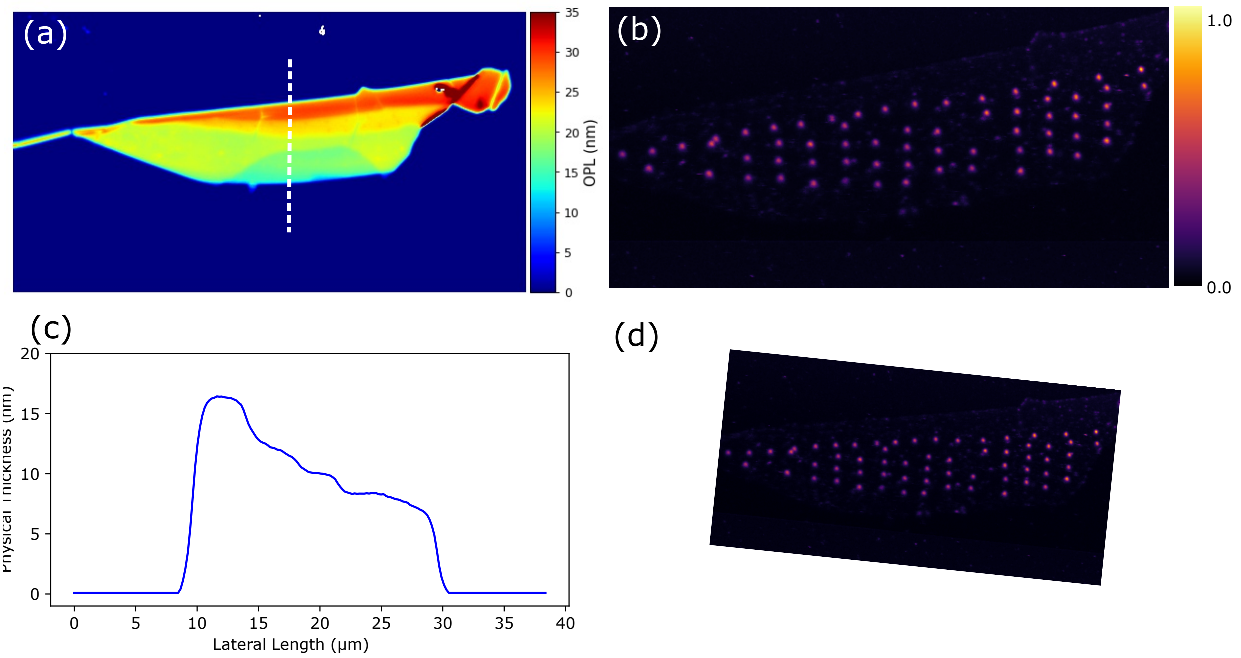

Figure S2: (a) A PSI map of the flake revealing the thickness profile. The color bar present the optical path length (OPL). (b) A PL map of the flake was collected using a 530 pulsed laser with a 20 MHz repetition rate. The bright spots correspond to the irradiated spots in the SEM image. (c) A thickness profile of the flake along the "white dash line" in (a). Here, OPL is converted into physical thickness corresponding to the actual thickness of the flake [1]. (d) The overlap of the thickness map and PL map of the flake correlates the distribution of bright emission in the PL map with the thickness of the flake.

S1.2 ZPL distribution

A histogram of the ZPL peak positions is extracted by measuring the emission of the various emitters exhibiting a $g^{(2)}(0)$ dip. Fig. S3 shows the histogram of ZPLs with a mean peak position at 574.84 nm and a standard deviation of 0.84 nm. Note that this includes spectra from other hBN flakes treated in the same way. The raw spectra included in the histogram are shown in the Fig. S5 recorded with a 530 nm pulsed excitation laser and a long pass filter with a cut-off wavelength of 550 nm.

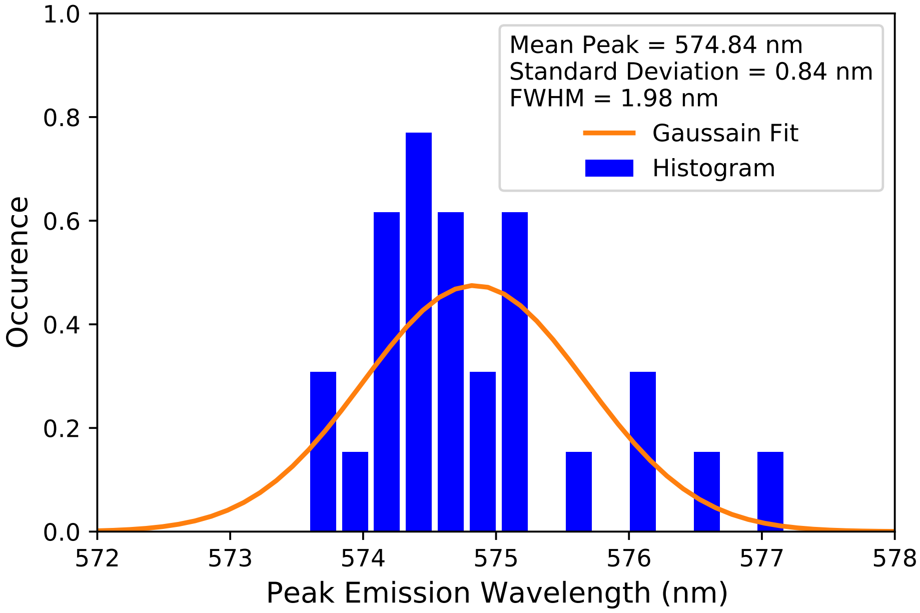

Figure S3: A histogram presenting the distribution of peak emission wavelengths of the emitters. Each emission spectra is collect using 530 nm pulsed excitation laser with 20 MHz repetition rate. The mean peak is at 574.84 nm with a standard deviation of 0.84 nm as extracted from the Gaussian fit to the histogram.

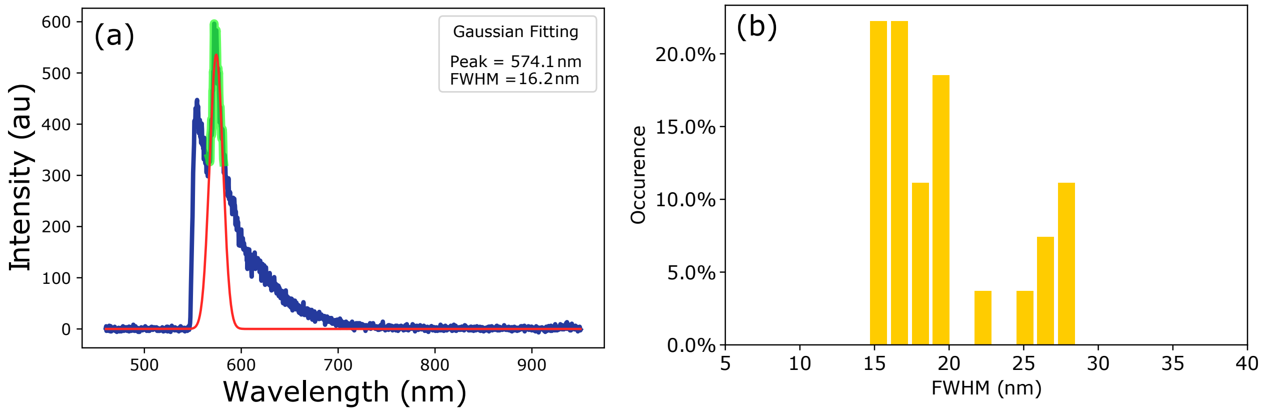

Figure S4: (a) A typical spectrum of the emitter. A Gaussian fitting is performed on the selected part of data. The estimated peak position is 574.1 nm and the FWHM of the peak is 16.2 nm. (b) The distribution of FWHM was extracted from the Gaussian fitting. The mean of the FWHM is around 19.56 nm with a standard deviation of 4.44 nm.

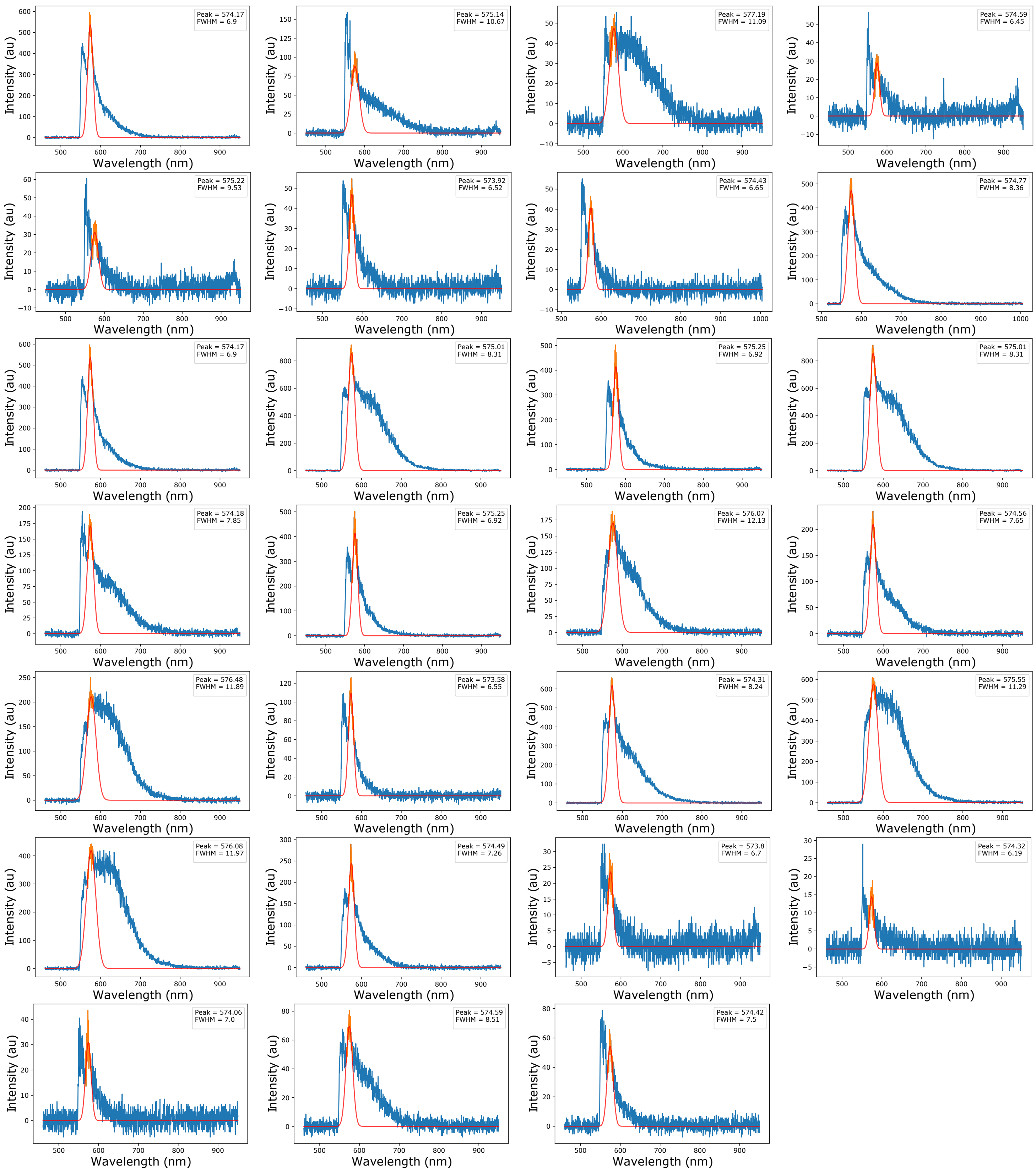

Figure S5: Spectra measured on different emitters exhibiting a g^2 dip using 530 nm pulsed excitation laser. A long pass filter with a cut-off wavelength at 550 nm is used to block the excitation laser.

S1.3 Emission spectra under 470 nm laser excitation

We observed that our spectrum has a sharp cut-off at 550 nm, with a peak at 575 nm under 530 nm pulsed excitation as shown in Fig. S6(top). We measured the spectrum at one of the irradiated

spots under 470 nm pulsed excitation laser as shown in Fig. S6(bottom). In this case, a long pass filter with cut-off wavelength of 500 nm is used to filter out the excitation laser. The spectrum does not reveal any sharp feature in the range of 500 nm to 550 nm, i.e., there is no new ZPL appearing. However, we see some peaks which are artifacts from the spectrometer and laser leakage, as these peaks are also present in the background spectrum. The background spectrum is collected on the hBN flake but away from the irradiated spot. The spectrum under 470 nm excitation laser suggests that there exists no zero phonon line in the spectral window blocked by the filter while using 530 nm excitation laser.

At the same time, we also notice we do not excite 575 nm peak. To understand it further, we mirrored the spectra shown in Fig. S7(top) collected using 530 nm pulsed excitation laser, around the ZPL peak at 575 nm. The mirrored spectra in Fig. S7(bottom) gives the absorption spectrum, i.e., the available phonon modes that the laser can excite into. We see a negligible overlap with the phonon side bands compared to the 530 nm excitation laser when excited using 470 nm excitation laser. Thus the 470 nm excitation laser has no phonon mode to excite the emitter into. We also note that we excited the emitter with a 375 nm laser and did not observe the 436 nm emitter [1].

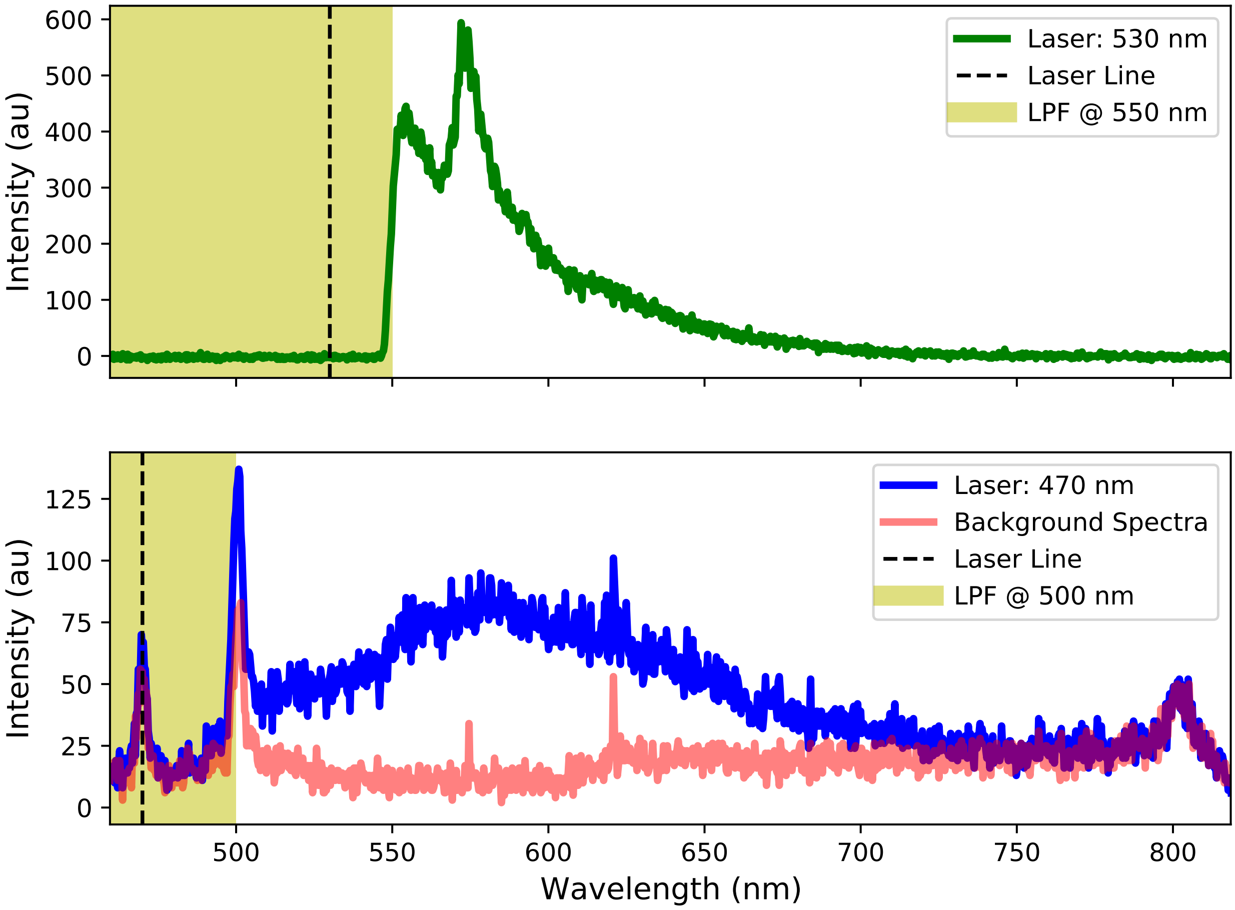

Figure S6: (top) Spectrum of emitter with 530 nm pulsed excitation laser, presenting a peak a 575 nm. (bottom) Spectra at one of the irradiated spot, collected with 470 nm pulsed excitation laser (blue). A background spectrum is also measured for the reference in red.

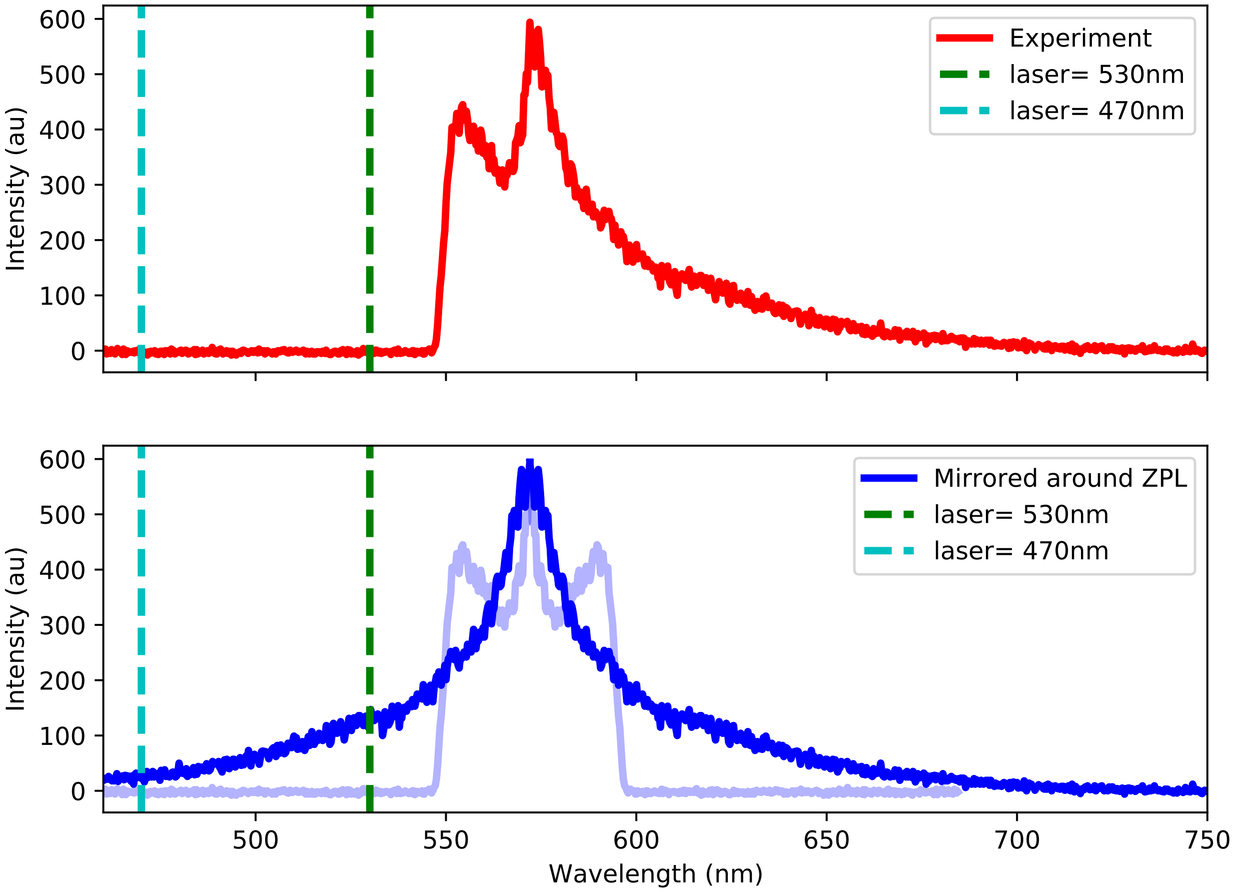

Figure S7: (top) Spectrum of emitter under the pulsed excitation laser of wavelength of 530 nm with repetition rate of 20 MHz. (bottom) Mirrored spectra around the ZPL at 575 nm presenting the overlap of side-bands with the excitation laser. The dashed lines present the excitation laser at 530 nm and 470 nm.

S1.4 Polarization-resolved second-order correlation measurements

The polarization resolved g^2 measurement selectively excite the emitter matching the emitter polarization and at the same time it does not excite other emitters close-by with a different polarization state. This leads to high purity of single photons and thus a high anti-bunching. The next section shows the polarization resolved correlation function measurements of the emitters in the array.

S1.5 $g^{(2)}$ distribution

All $g^{(2)}$ functions were recorded using 530 nm pulsed excitation laser with the repetition rate of 20 MHz.

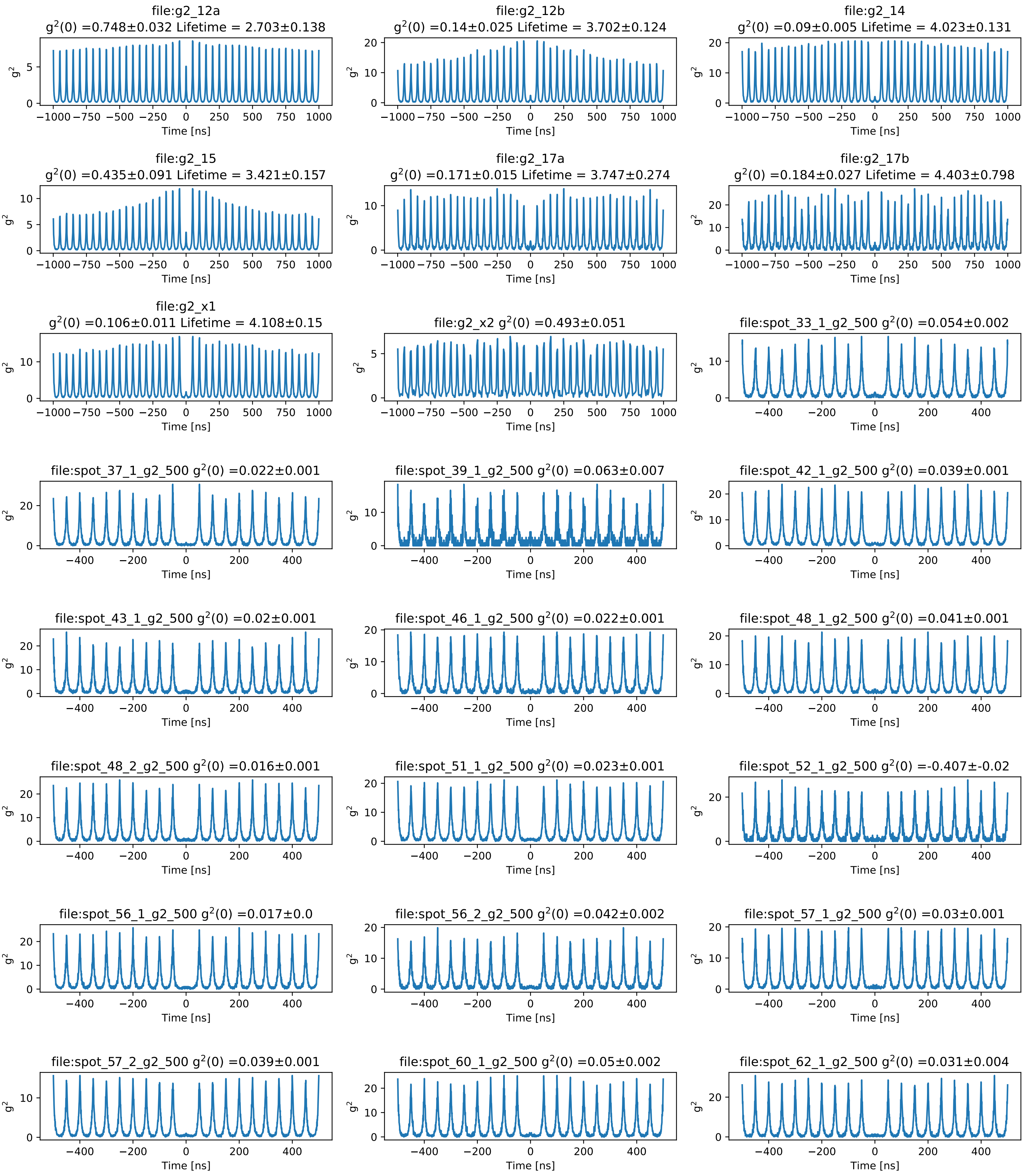

Figure S8: Normalized g^2 curves corresponding to the data points in Figure 2 (main manuscript). Note that the error bars from the g^2 normalization fit is <5%. We also extracted the lifetime for some emitters from the g^2 fit as mentioned in the plot title. Note that all the g2 measurements are carried out using 530 nm pulsed excitation with a repetition rate of 20 MHz.

S1.6 Lifetime distribution

All the lifetime decay curves were recorded using 530 nm pulsed excitation laser with repetition rate of 20 MHz.

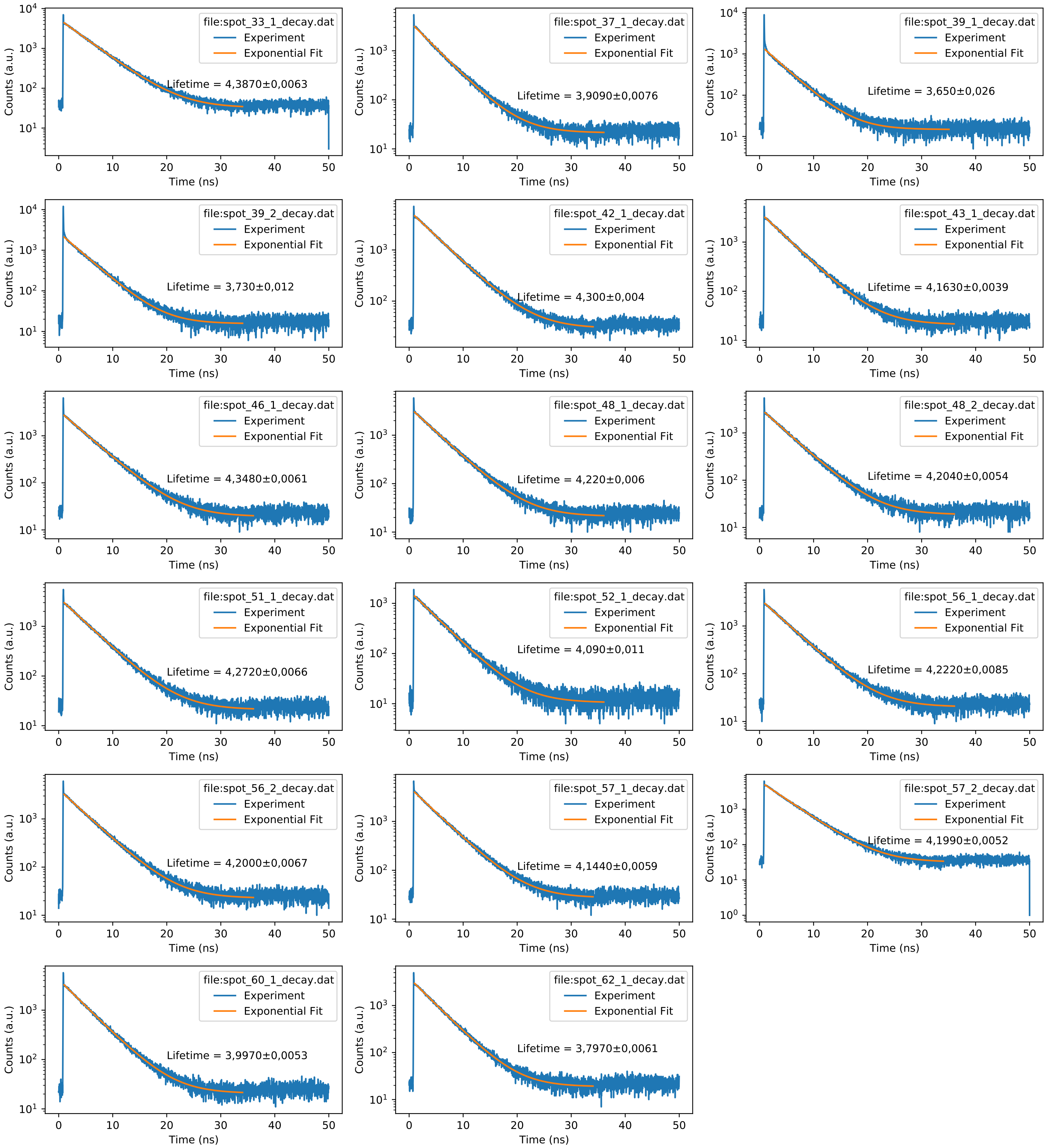

Figure S9: Lifetime decay curve of the various emitters in the figure-2 recorded using 530 nm pulsed excitation laser with 20 MHz repetition rate. A bi-exponential fitting function is used to estimate the lifetime of the emitter.

S2 Polarization dynamics data acquisition

The optical characterization of the emitters was performed on a commercial fluorescence lifetime imaging microscope (PicoQuant Micro-Time 200). To perform polarization-resolved measurements, we extended the capabilities of the instrument by inserting polarising elements in the setup, which include: (i) a fixed linear polarizer in the excitation laser path to reset the polarization to horizontal with a high extinction ratio. (ii) A quarter-wave plate that creates circularly polarised light. (iii) A polarizer that scans the emitter excitation axis after the quarter-wave plate. (iv) A polarizer in the detection path while measuring the emission polarization. All these elements were mounted on motorized rotation mounts (Thorlabs ELL14). The rotation mounts were interfaced using a self-developed python script using a portable PC.

S2.1 Creation of circular laser polarization

Laser light in the excitation path is first converted into circularly polarized light. To create circular polarization, a linear polarizer is placed in the path of a fiber-coupled laser and afterwards a quarter wave plate is placed in the path. The quarter-wave plate's fast axis is aligned at an angle of 45° with respect to the transmission axis of the polarizer. This combination yields a more accurate polarization setting compared to using a simple half-wave plate. The latter is only available for specific wavelengths (nearest to the laser is 532 nm) which can result in a slight offset in the actual polarization setting. The actual excitation polarization is then set by another linear polarizer. Fig.S10 shows the laser power variation as a function of the rotation angle of this polarizer. The degree of linear polarization is below 5%, indicating a well circularly polarized laser. This results in a nearly independent laser power (of the polarization). The degree of linear polarization is calculated using fitting

$$I(\theta) = a \cdot \cos^2(\theta - b) + c, \tag{1}$$

where we extracted $a = 4.53\ \mu$W, $b = 178.24°$, and $c = 46.57\ \mu$W. The degree of (linear) polarization is defined as

$$\mathrm{DOP} = \frac{\mathrm{I_{max}} - \mathrm{I_{min}}}{\mathrm{I_{max}} + \mathrm{I_{min}}} = \frac{51.10 - 46.70}{51.10 + 46.70} \approx 0.05 \tag{2}$$

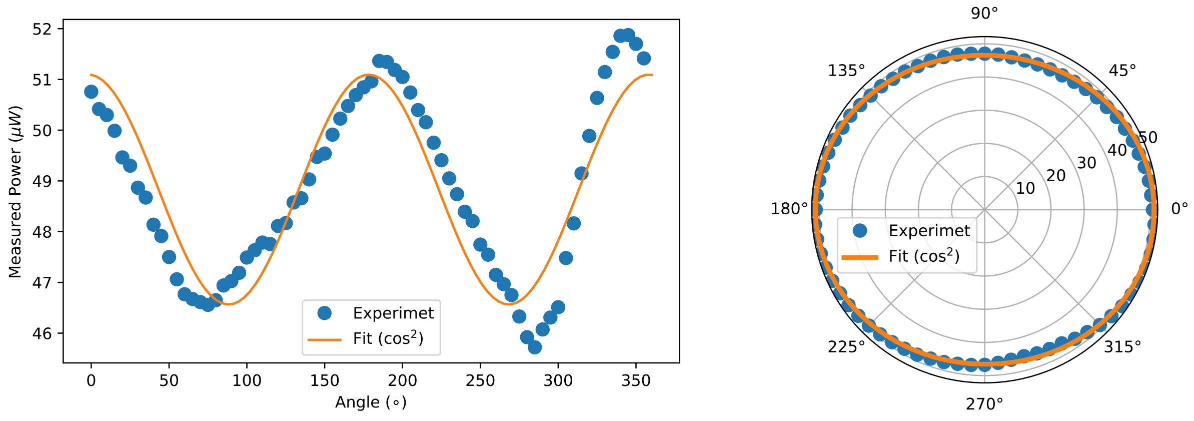

(a) (b)

Figure S10: (a) Linear and (b) polar plot of the excitation laser power as a function of the rotation angle of the polarizer. The excitation wavelength of the pulsed laser is 530 nm with a repetition rate of 20 MHz. A cosine squared function is used to fit the data. The degree of polarization is estimated to be around 5%.

S2.2 Fitting Function

In principle, we employed the same fitting function as in Eq. 1 to fit the dipole polarizations. However, we observed a small shift in our scanning stage which becomes relevant in measurements

taking a long time. This results in the sample running out of focus over time. We found this shift to be linear (in time, i.e., also in angle for equidistant time steps per rotation step), which allows us to easily compensate for this using the following adapted fitting function:

$$I(\theta) = a \cdot (1 - b \cdot \theta) \cdot \cos^2(\theta - \phi) + c, \tag{3}$$

where a, b, c, and ϕ are the fitting parameters. The parameter ϕ is the relevant polarization axis.

S2.3 Excitation polarization

The excitation axis of polarization is measured using the linear polarizer after creating the circular polarization. We created PL maps of the emitters for every rotation step, as shown in Fig. S11. This is necessary due to the laser spot shifting relative to the fixed emitter position during the rotation of the optic, which also affects the count rate at the detector. To avoid this laser misalignment, we create photo-luminescence (PL) maps covering the larger scan area. The dwell time per pixel is set to 2 ms. Later in the data analysis, we calculated the emitter's integrated PL intensity over a diffraction-limited area as shown in the Fig. S11 by the red box. Similarly, we also extracted the integrated background intensity denoted by the yellow box. Note that while creating these PL maps, there is no polarizer in the detection path. The extracted count rate from an emitter is shown in Fig. S12. A cosine squared function as in Eq. 1 is used to fit the experimental data to extract the degree of polarization and axis of polarization.

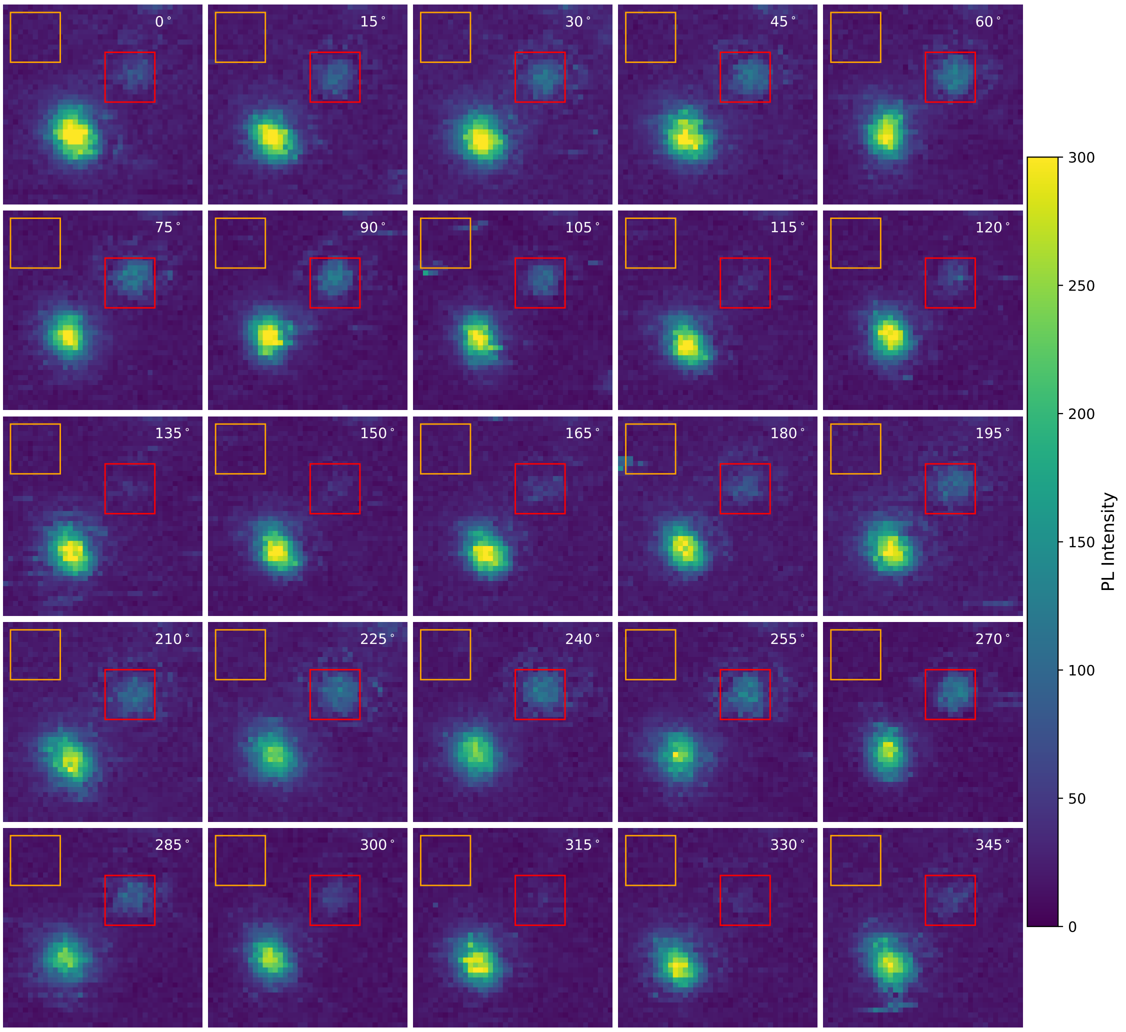

Figure S11: The PL maps of the emitter at every rotation step (15° increment) of the polarizer in the excitation path. A 530 nm pulsed excitation laser is used to record every PL map with the average power of the laser being fixed around 50 μW. The integrated PL intensity of the emitter is extracted from the red box and the integrated background intensity is also extracted from the yellow box in the PL maps.

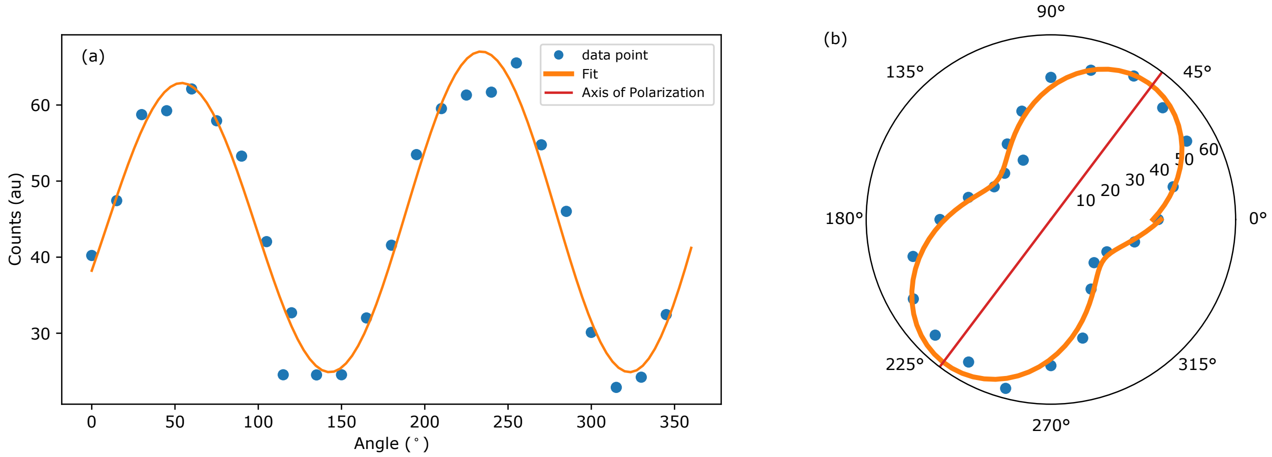

Figure S12: (a) Linear and (b) polar plot of the resulting integrated photon count rate extracted from the PL map as a function of the rotation angle in the excitation path. A cosine squared function is used to estimate the polarization axis and polarization visibility.

S2.4 Emission polarization

The emission axis of the polarization is measured using a polarizer in the detection path. We recorded the time trace (signal counts over time) while rotating the polarizer in the detection path. The dwell time for each rotation step is chosen to be 5 or 10 s. The count rate is logged every 100 ms. Before recording the time trace for the emitter, the polarizer in the excitation path is optimized to efficiently excite the emitter, which maximizes the PL signal in the detection path. A typical time trace is shown in Fig. S13(a). The step-like change in the PL intensity is observed in the time trace at the interval of the set dwell time. The mean count is calculated in between the red and green dashed lines to find the count as a function of the angle. Fig. S13(b) shows the extracted mean PL count from the time trace. Afterwards, the extracted data is fitted using the cosine squared function in Eq. 1 to find the axis polarization and the degree of polarization as shown in Fig. S13(c) and (d), respectively.

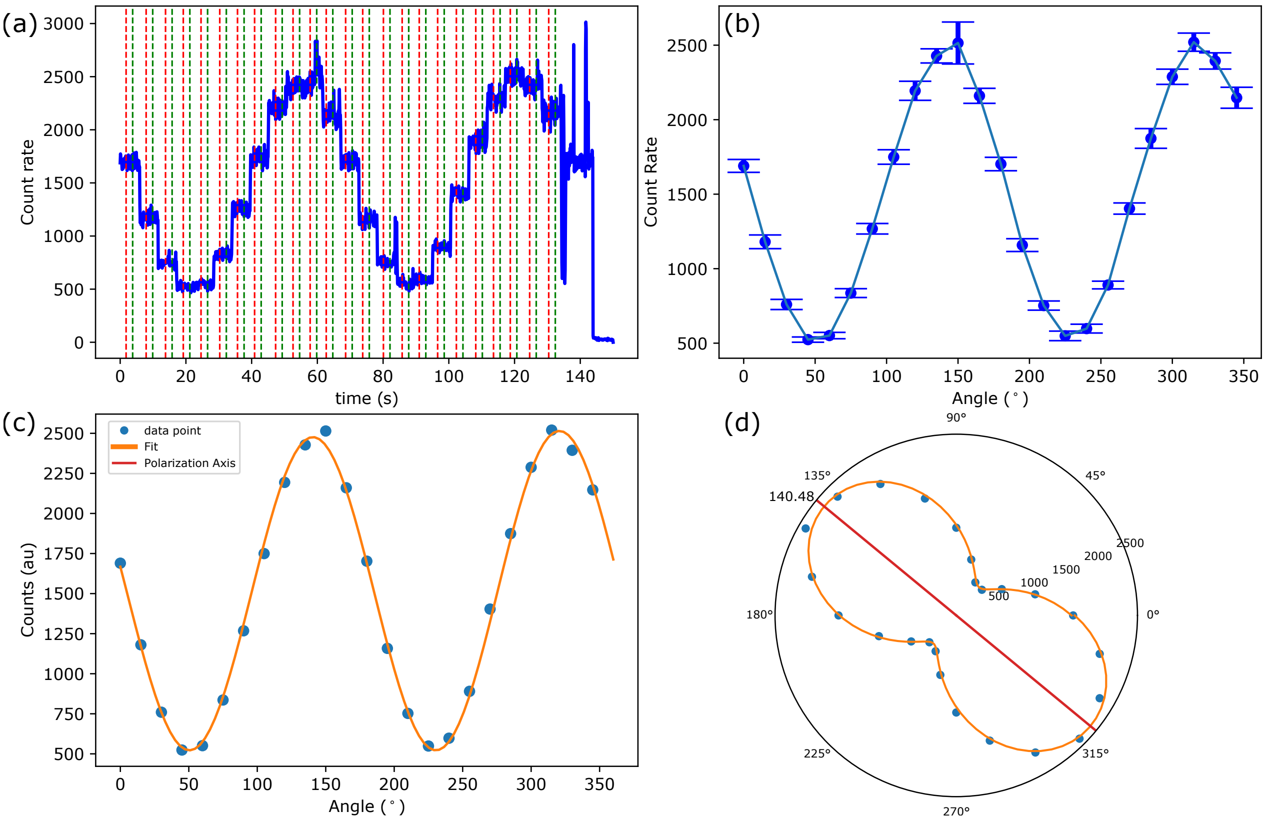

Figure S13: The emitter is excited using a 530 nm pulsed excitation laser with a repetition rate of 20 MHz.(a) Time trace recorded with a polarizer rotation dwell time of 5 s. The red and green dashed lines indicate the data used to calculate the mean and standard deviation. Sufficient time for the step motors to come to rest is accounted for. (b) The mean count extracted from the time trace plotted against the rotation angle of the polarization in the detection path. (c) Linear and (d) polar plot of the extracted raw data and fitted with the cosine squared function.

S3 Polarization dynamics data analysis

S3.1 Excitation and emission polar plots of the emitters

Each plot in Fig. S14 is recorded using a 530 nm pulsed excitation laser. The axes of polarization and polarization visibility is estimated from the cosine squared fitting function given in Eq. 2 and 3.0 The error for each parameter are extracted from the fit.

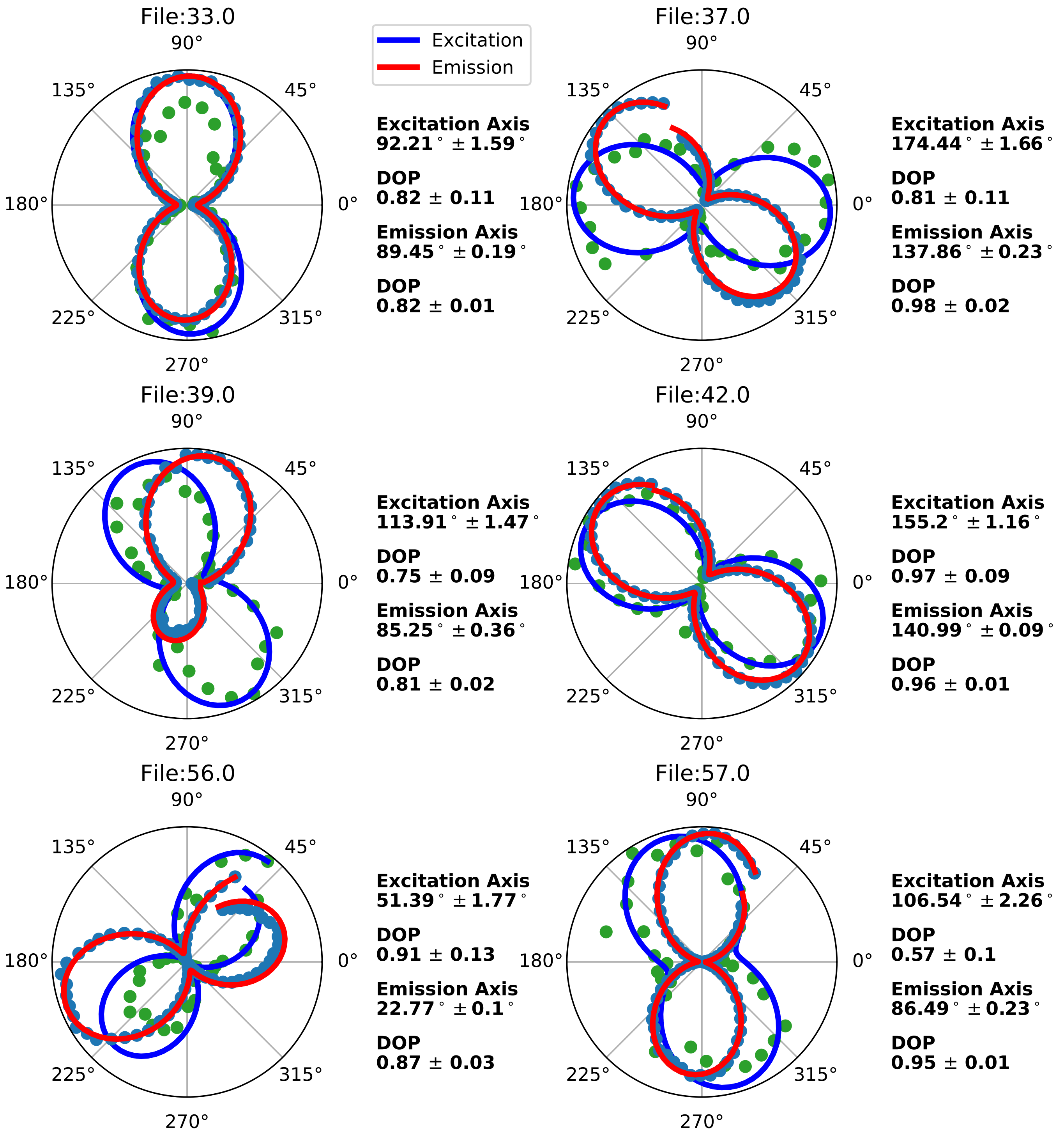

Figure S14: Excitation and emission polar plots of the various emitters investigated in the Figure 2 of the main manuscript. A cosine fitting function is used to extract the polarization axes and degree of polarization (DOP) and corresponding error bars.

S3.2 Dipole correlation with flake thickness

A multi-layer hBN flake is used for the experiments. The contrast-enhanced optical microscope image of the flake is shown in Fig. S15(a). The different colors represent different thicknesses of the flake. Fig. S15(b) shows the corresponding PL map of the flake, where the green and yellow arrows present the direction of excitation/emission axes of polarization respectively. When these images are overlayed with a 60% transparency level as shown in Fig. S15(c), the dipole orientation

can be correlated with flake thickness. As can be seen, the emitters appear uniformly throughout the flakes, independent of the flake thickness. This suggest that there is no correlation between the flake thickness and the occurrence of the emitters.

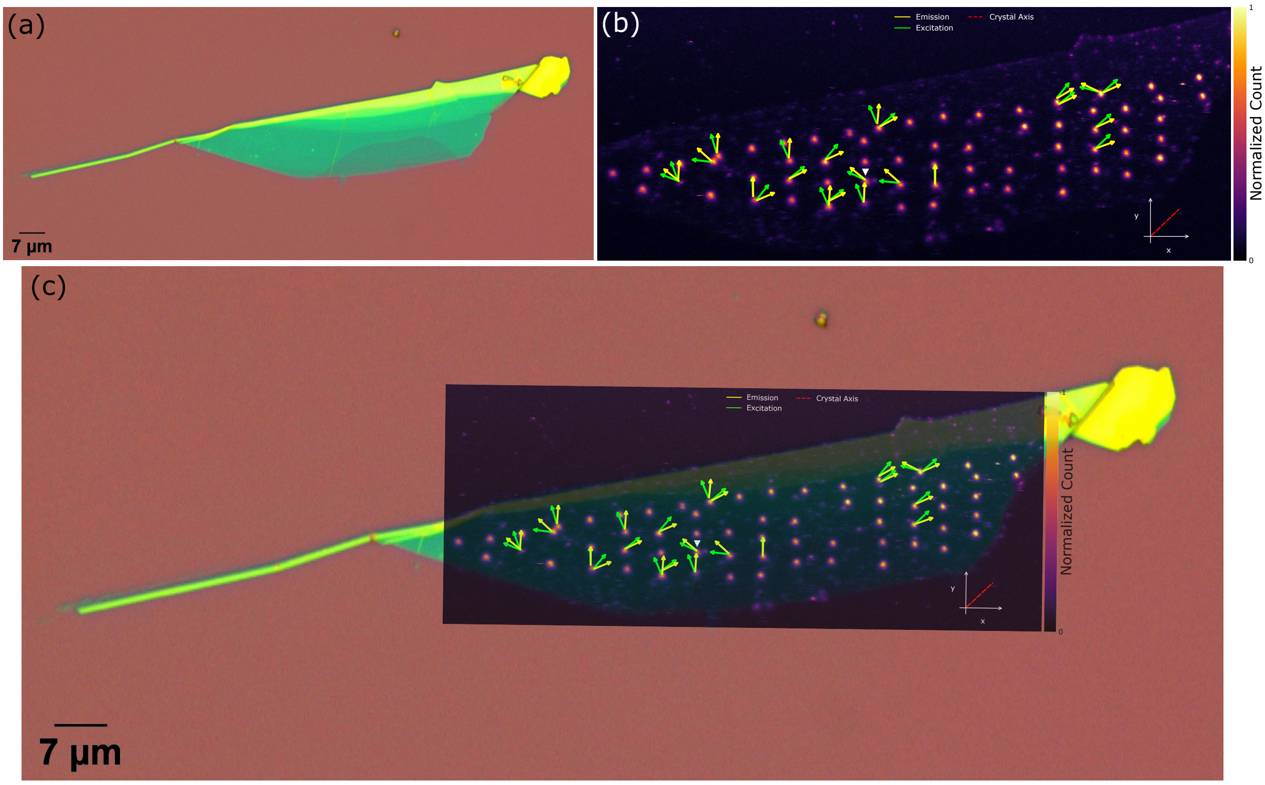

Figure S15: (a) Contrast-enhanced bright field optical image of an hBN flake used to fabricate emitter using localized electron irradiation. (b) A PL map of the flake recorded using a 530 nm pulsed excitation laser. The green and yellow arrows denote the excitation and emission axes of polarization respectively. (c) An overlayed image of the microscope image and PL map of flake reveals no correlation between dipole axis and the flake thickness.

S4 Second-harmonic generation measurement

Second-harmonic-generation (SHG) measurements were performed in a reflection geometry as shown in Fig. S16(a). The hBN flake is first positioned in the focal point of the 50× objective (0.55 NA) using a white light source for illumination and a high-sensitivity thermo-electrically cooled CCD (see Fig. S16(b) and (c)). An 800 nm wavelength mode-locked Ti:Sapphire laser with 76 MHz repetition rate, 200 fs pulse duration (estimated at the sample position) and an average power of 20 mW is used for pumping the SHG process. The pump is focused onto the hBN flake and the polarization is set with a motor-controlled half-wave plate. The generated second-harmonic light at 400 nm wavelength is collected by the same objective and separated from the pump by a 50:50 beam splitter. A motor-controlled polarizer is used after the beam splitter to control the measurement angle, and spectral filters are used to remove any residual pump light. The same high-sensitivity CCD is used to record the SHG signal, where the pixels at the excitation location are integrated to give a count rate. The SHG is initially verified by the quadratic dependence on pump power (see Fig. S16(d)) and secondly by the polarization dependence (see Fig. S16(e) and (f)).

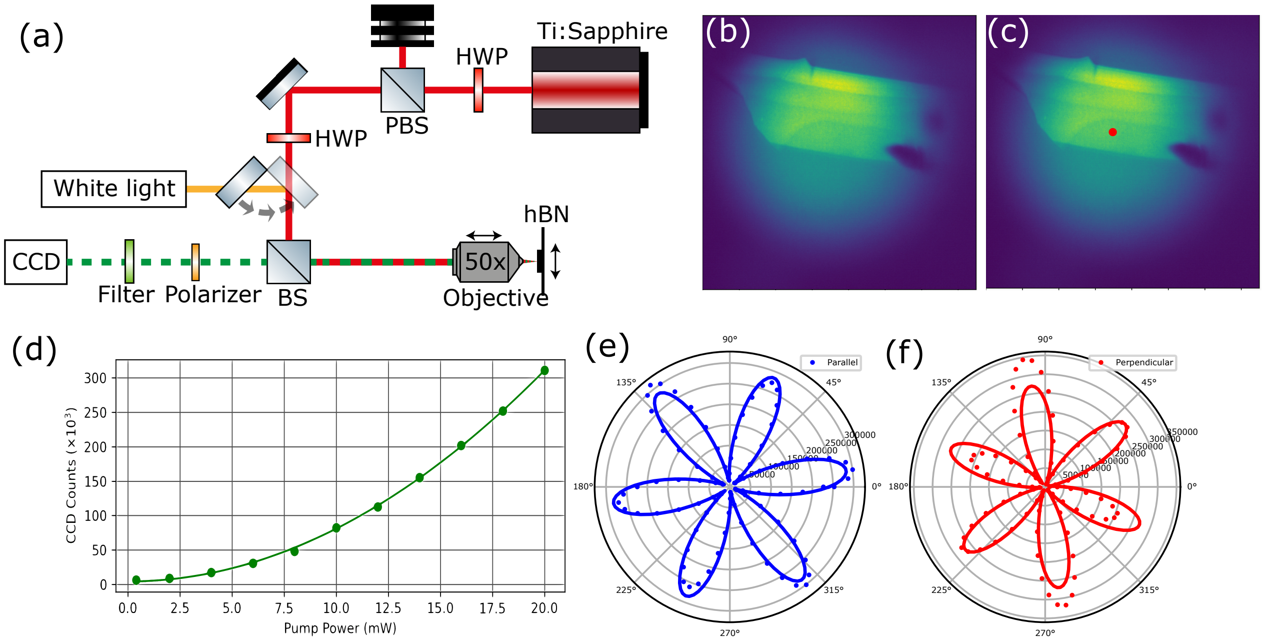

Figure S16: (a) The experimental setup for the SHG measurement. (b) The optical image of flake under the SHG setup. The position of the laser spot is marked with red dot in (c). (d) The SHG signal is recorded as a function of pump laser power, fitted with a quadratic relation, confirming that the detected signal is from an SHG process. (e) and (f) Polar plots of SHG signal as a function of the rotation angle with laser being parallel and perpendicular, respectively.

S5 Misalignment between excitation, emission axis and crystal axis

The statistics of the excitation and emission dipoles (here we mean the excitation and emission polarization directions) are shown in Fig. S17. As can be seen, these dipoles bunch in groups. The crystal axis is marked with red and blue dashed lines as determined by the SHG experiment.

The center of the groups for excitation aligns well with the crystal axes within a few degrees, however, the standard deviation within these groups is considerably larger ranging from 5°-10° (see Fig. S17(a)).

For emission, this standard deviation is much smaller (within a few degrees), but the distance from the crystal axes ranges from 16° to 21° (see Fig. S17(b)). In addition, the mean values are not separated by 60°, which would be expected in an hexagonal crystal. When the difference between the excitation and emission angle is calculated, this results in a large standard deviation as well (see Fig. S17(c)). The mean value of the difference here is 18.89° with a standard deviation of 10.07°.

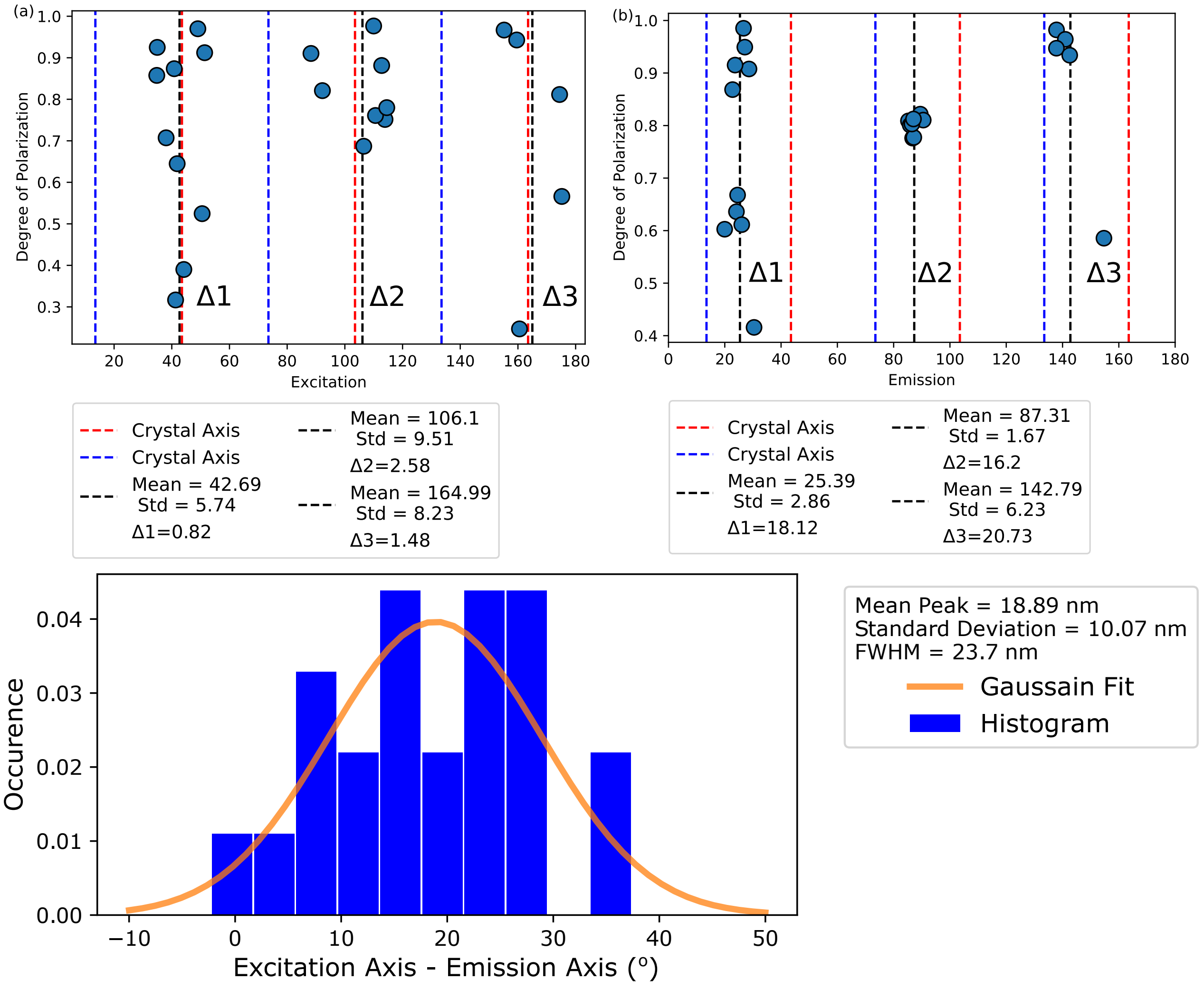

Figure S17: Statistical distribution of (a) excitation and (b) emission axes with the crystal axes marked by the dashed lines (red denotes the direction along the bonds and blue perpendicular to the bonds). The black dashed lines present the mean of the angles of each group and [Δ1, Δ2, Δ3] present the misalignment between crystal axis and mean of each group. (c) The histogram showing the misalignment between excitation and emission polarization axes. The extracted mean of 18.89° with a standard deviation of 10.07°.

S6 Temporal polarization dynamics

Fig. S18 displays the post-process algorithm that we have used to analyze the polarization dynamics of the quantum emitters. The top left figure is the polarization-resolved emitter lifetime map that we have created by stacking decay measurements at different polarization angles. The steps we have used from there are described below.

- Step 1 - Polarization-resolved decay map is divided into N time bins, where N is related to desired temporal resolution for the analysis and the total temporal range that we want to analyze.
- Step 2 - A loop is iterated over all time bins. Loop iteration count "i", starts from 1 and goes to N, where N is the last time bin.
- Step 3 - For every i-th time bin, the segment is isolated and integrated in time axis, then a cosine squared function is fitted to the data.
- Step 4 - For that i-th time bin, the orientation is extracted from the fit function, and linear

polarization visibility is calculated from the fit parameters.

- Step 5 - Finally, all visibility and orientation values of different time bins are combined.

Fig. S19(a) shows a density plot of the polarization-resolved emitter lifetime. To estimate the degree of polarization and axis of polarization, we fitted again a cosine-squared relation through the time slices. The extracted and fitted data is shown in Fig. S19(b). From these fits, we can simply extract the polarization angle and degree of polarization as a function of time after the laser pulse (see Fig. S19(c) and (d), respectively.

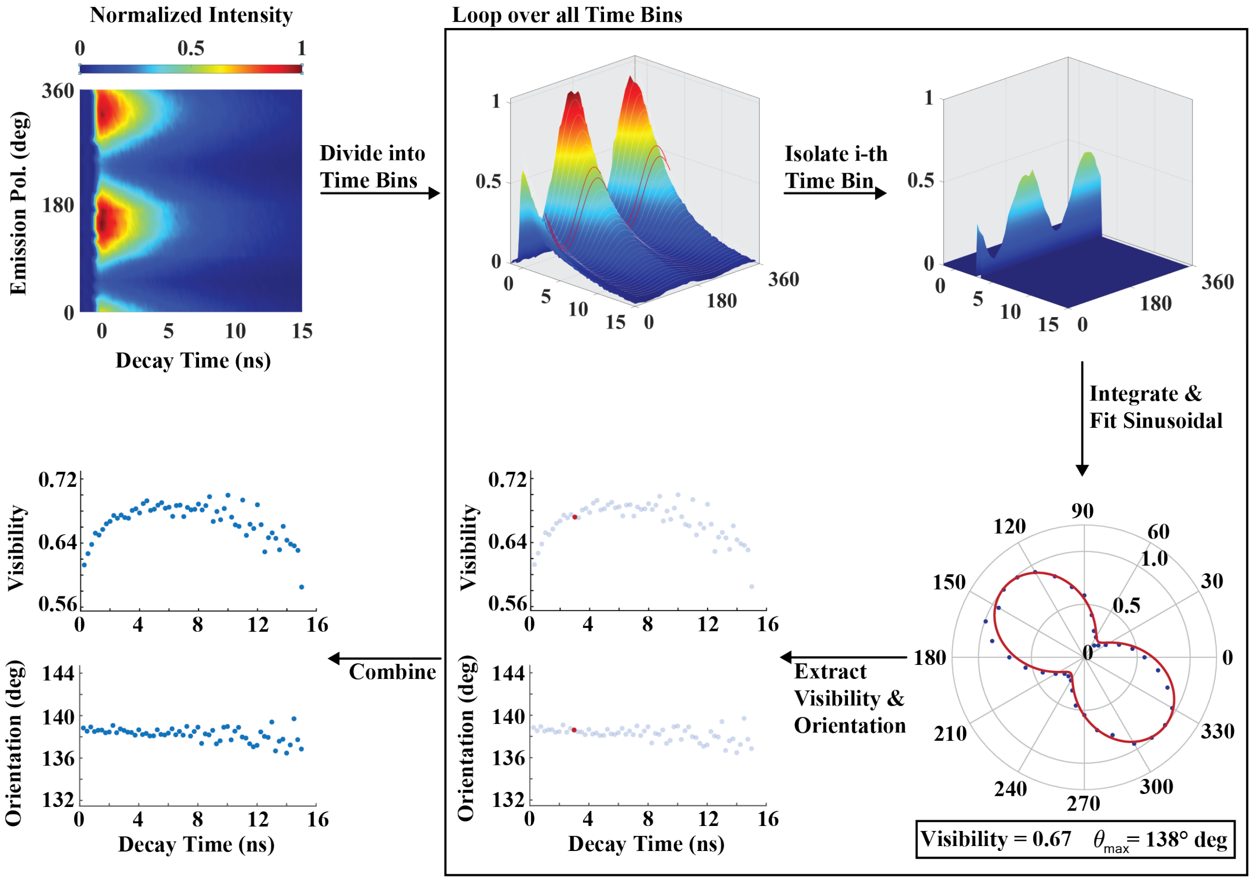

Figure S18: Post-process algorithm to extract dynamic evolution of polarization of quantum emitters. Polarization resolved decay map (top left figure) is composed by combining decay measurements at different polarizer angles.

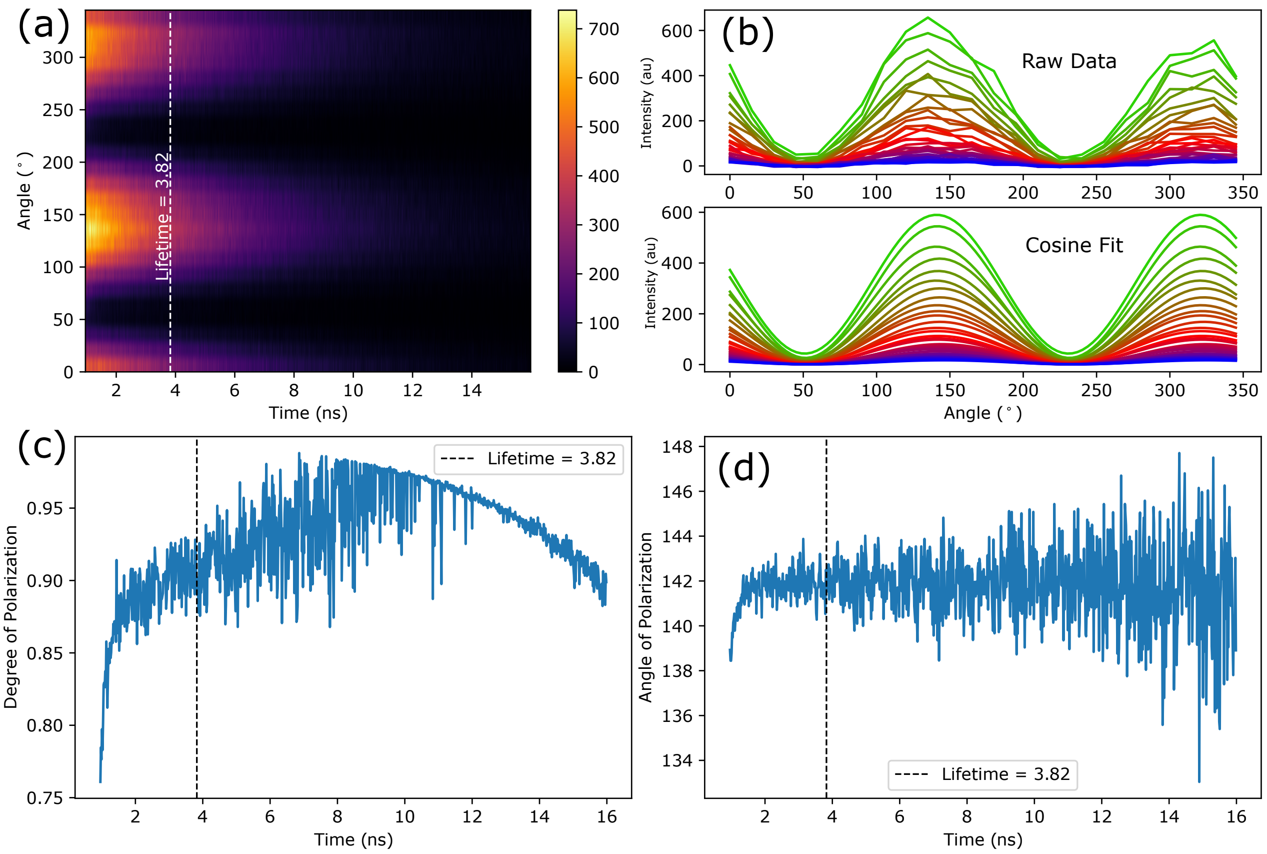

Figure S19: (a) Experimentally measured lifetime decay map with the rotation angle of the polarizer in detection path. (b) Cosine-squared fitting on the raw data is performed for all time slices. For clarity, only every 33rd slice is shown. The degree of polarization is estimated in (c), while the axis of polarization is estimated in (d) from the fits (in both cases).

To further study the temporal polarization of the emitters, we fitted the time dependent degree of polarization and axis of polarization using an exponential function in Eq. 4:

$$f(x) = a \cdot e^{p \cdot \tau \cdot x} + c. \tag{4}$$

Here, a and c are general fitting parameters dependent on the setup, p is a phase sign (± 1) and τ is the decay constant. The degree of polarization increases before reaching to the steady state and the estimated time from fit is found to be in the range of 0.5 to 1 ns. In the same way, the axis of polarization changes on a similar timescale. More statistics and measurements on the different quantum emitter systems can be found in the following. We have investigated irradiated hBN emitters in multilayer flakes, natural hBN emitters hosted in nanoflakes, and NV centers in diamond.

S6.1 Sample-1: irradiated hBN emitters

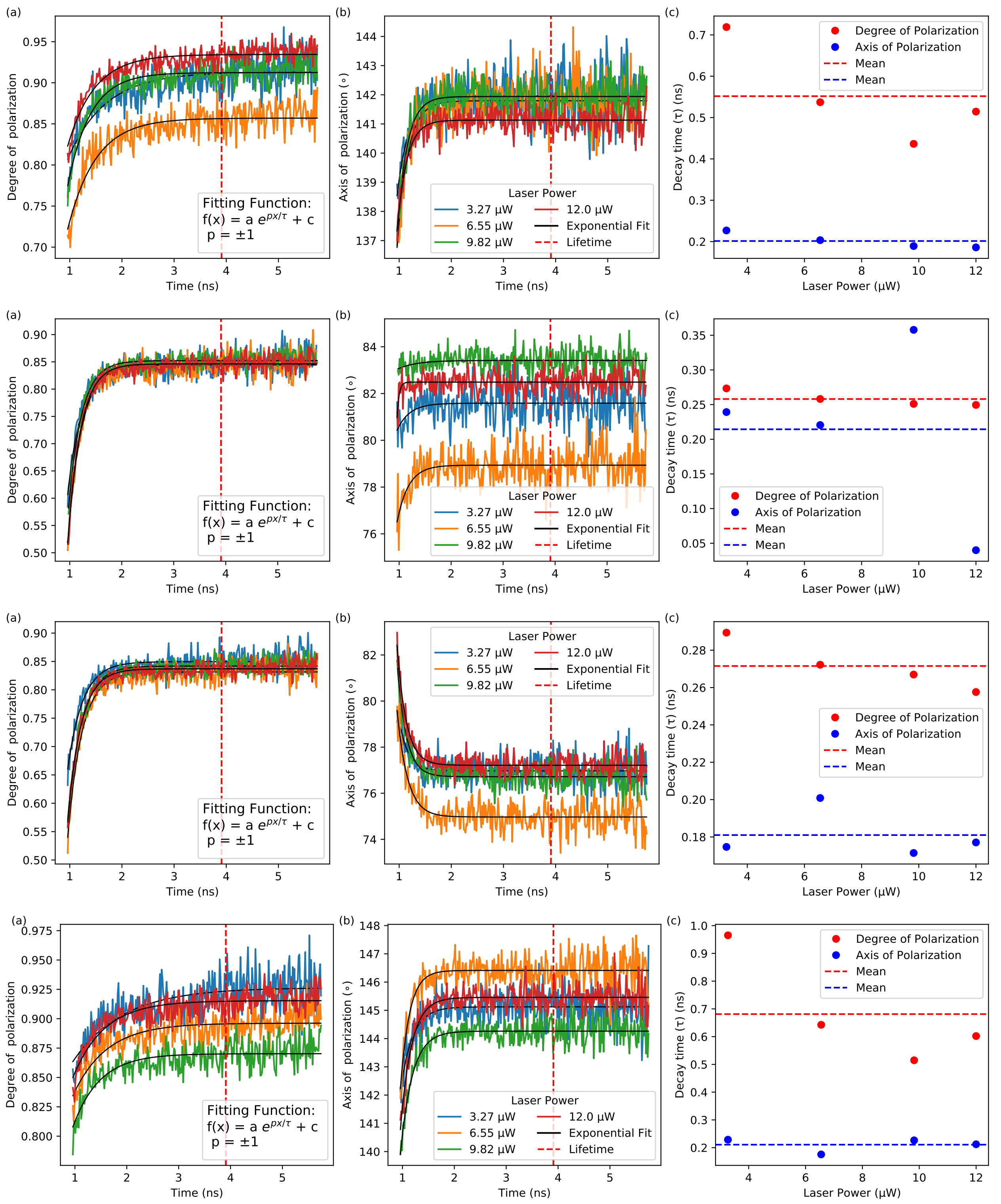

Figure S20: (a) Degree of polarization and (b) axis of polarization as function of time after the laser pulse for four different emitters (top to bottom) and resolved at different laser powers. (c) The parameters of the fitted decay parameters as a function of laser power reveals no dependency.

S6.2 Sample-2: nanoflake hBN emitters

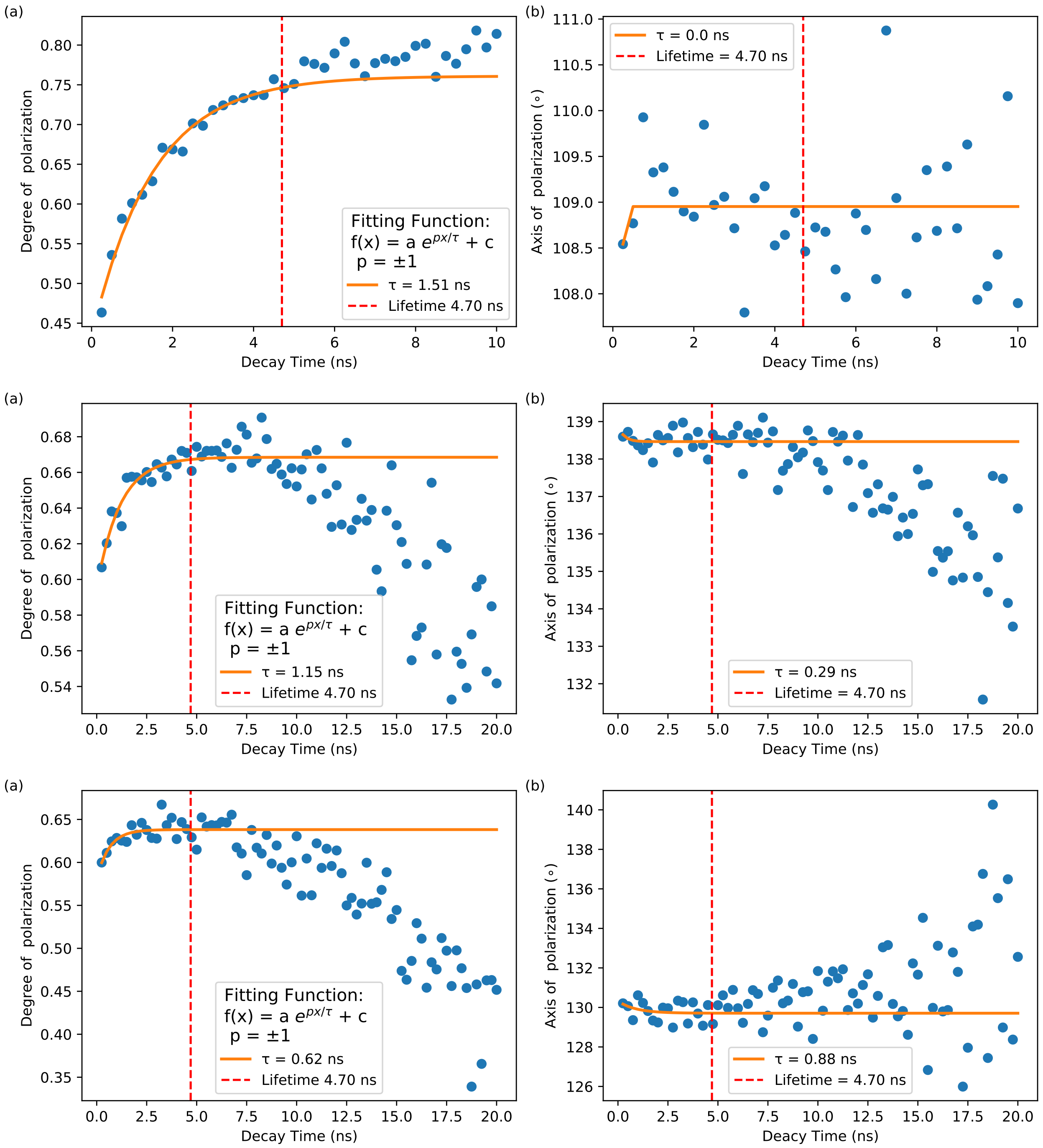

Figure S21: (a) Degree of polarization and (b) axis of polarization as function of time after the laser pulse for four different emitters (top to bottom) and resolved at different laser powers.

S6.3 Sample-3: NV centers in diamond

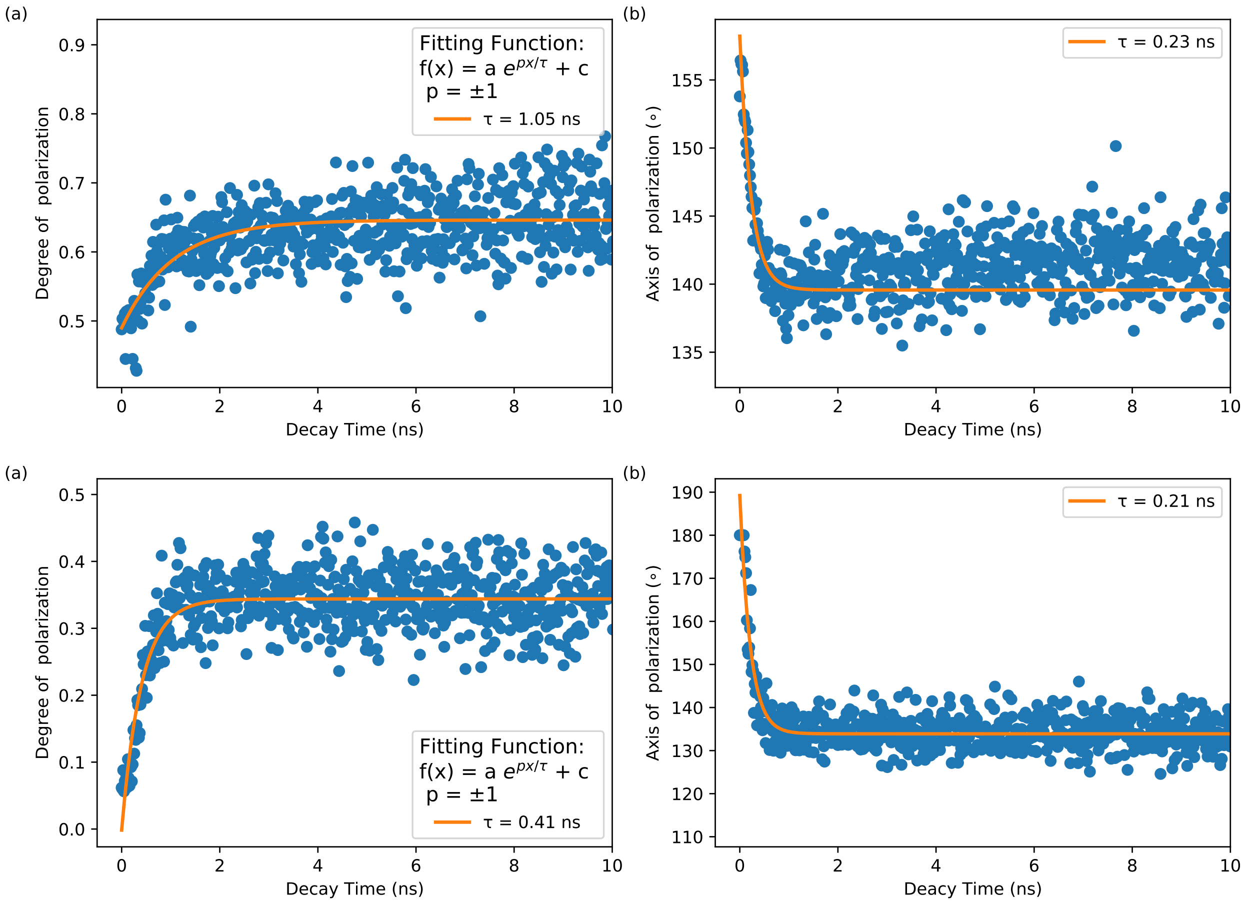

Figure S22: (a) Degree of polarization and (b) axis of polarization as function of time after the laser pulse for four different emitters (top to bottom) and resolved at different laser powers.

S7 hBN nanoflake quantum emitters

An optical investigation of hBN nanoflakes was performed using a custom-built confocal microscope setup, as shown in Fig. S23. The excitation port includes a pulsed excitation laser emitting at 483 nm with a 50 ps pulse length, along with a half-wave plate to maximize the excitation efficiency of the emitters. A 550 nm long pass dichroic mirror is used to direct the laser into the objective lens and filter the reflected laser from the sample, with an additional long pass or notch filter to fully suppress the laser. The emission path includes a half-wave plate and a polarizing beam splitter to study the polarization dynamics of the emitters. In the detection port, spectral features are investigated by a CCD camera attached to a spectrometer, and time-correlated single photon counting (TCSPC) measurements are performed at the side exit of the spectrometer. Finally, a Hanbury Brown and Twiss (HBT) interferometer is integrated into a side exit of the spectrometer to measure the second-order photon correlation of the emitters.

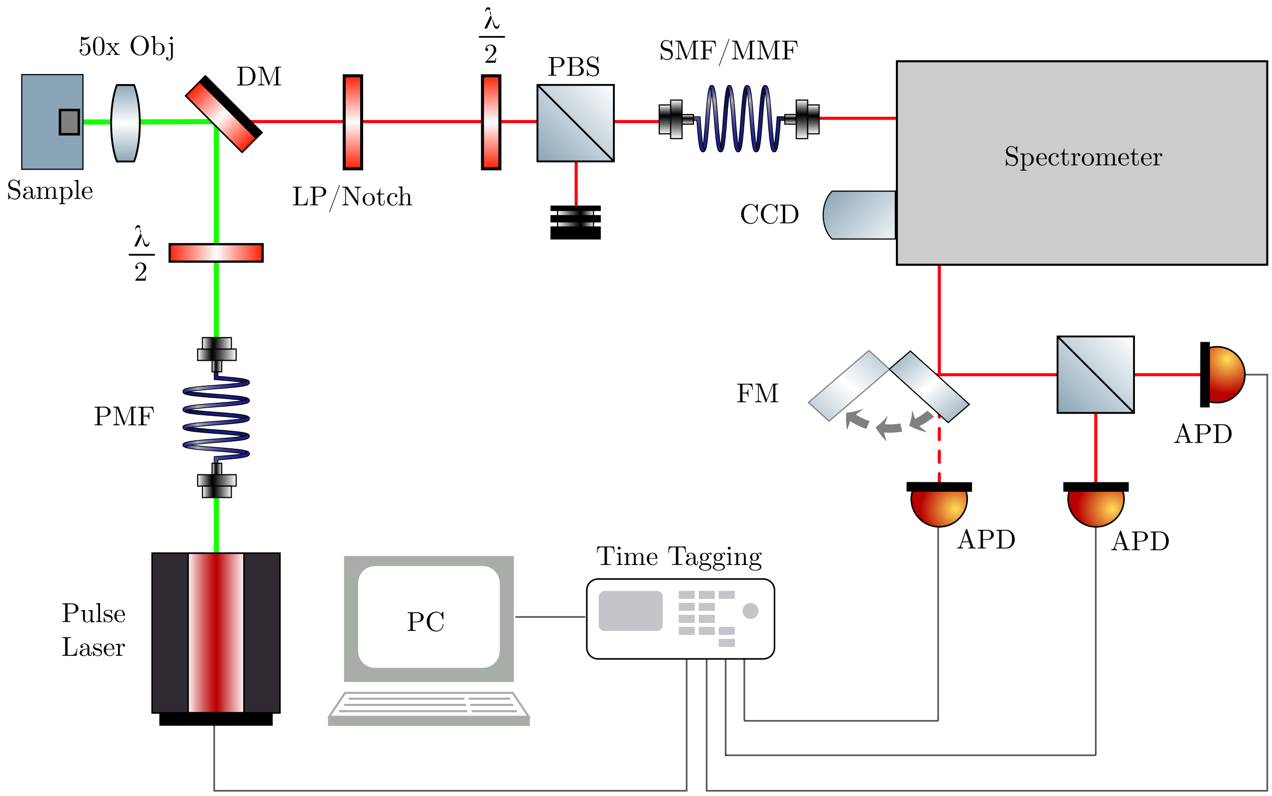

Figure S23: The experimental setup used for optical characterization of the quantum emitters hosted by hBN nanoflakes including the temporal polarization measurements. The setup comprises a 483 nm pulsed laser with a 50 ps pulse length, a high numerical aperture objective lens with 0.9 numerical aperture and 50× magnification, a spectrometer with a CCD camera, and avalanche photodiodes (APDs) for TCSPC measurements in the detection path.

The general photophysical properties of the emitters as well as their temporal polarization dynamics is shown in Fig. S24, S25, and S26.

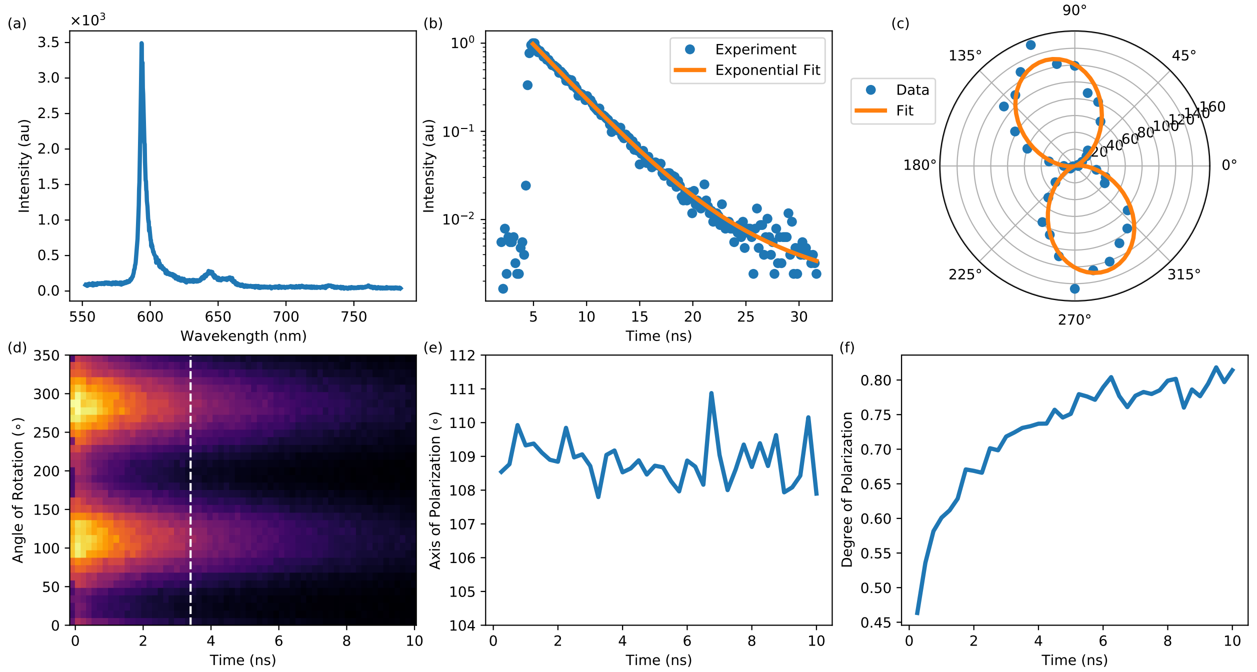

Figure S24: (a) Photoluminescence spectrum of an exemplary hBN nanoflake defect emitting at 594 nm, tagged as Emitter-1 in the main text. (b) Time-resolved PL intensity of Emitter-1 with a decay time of $\tau = 3.5$ ns extracted from the fit. (c) Emission polarization-dependent PL intensity, indicating a polarization angle of 109∘, extracted from the fit function. (d) The PL intensity of Emitter-1 as a function of the rotation angle of the polarizer and decay time, where each row (horizontal data) represents a time-resolved PL intensity measurement at the given polarization angle. (e) Axis of polarization as a function of time after the laser pulse of the Emitter-1, showing small fluctuations around 109∘ with no significant deviation. (f) Visibility of linear degree of polarization as a function of decay time. For figures (e) and (f), 200 picosecond time bins are used.

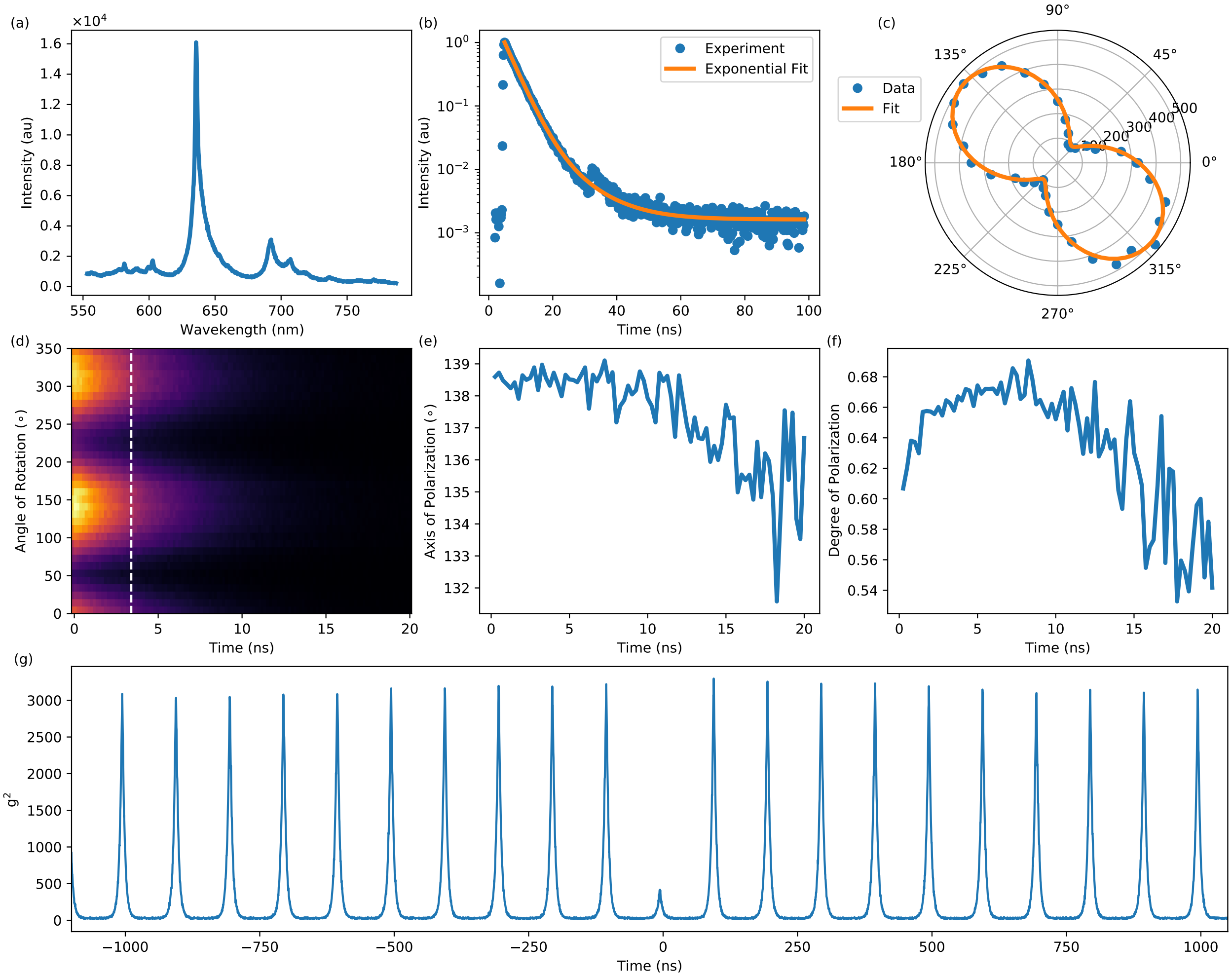

Figure S25: (a) Photoluminescence spectrum of an exemplary hBN nanoflake defect emitting at 636 nm, tagged as Emitter-2 in the main text. (b) Time-resolved PL intensity of Emitter-2 with a decay time of $\tau = 3.6$ ns extracted from the fit. (c) Emission polarization-dependent PL intensity, indicating a polarization angle of 141$\circ$, extracted from the fit function. (d) The PL intensity of Emitter-2 as a function of the rotation angle of the polarizer and decay time. (e) Axis of polarization as a function of decay time of the Emitter-2. (f) Visibility of linear degree of polarization as a function of decay time. For figures (e) and (f), 200 picosecond time bins are used. (g) Second-order correlation measurement of the emitter. Anti-bunching at the zero delay indicates the single quantum nature of the emitter.

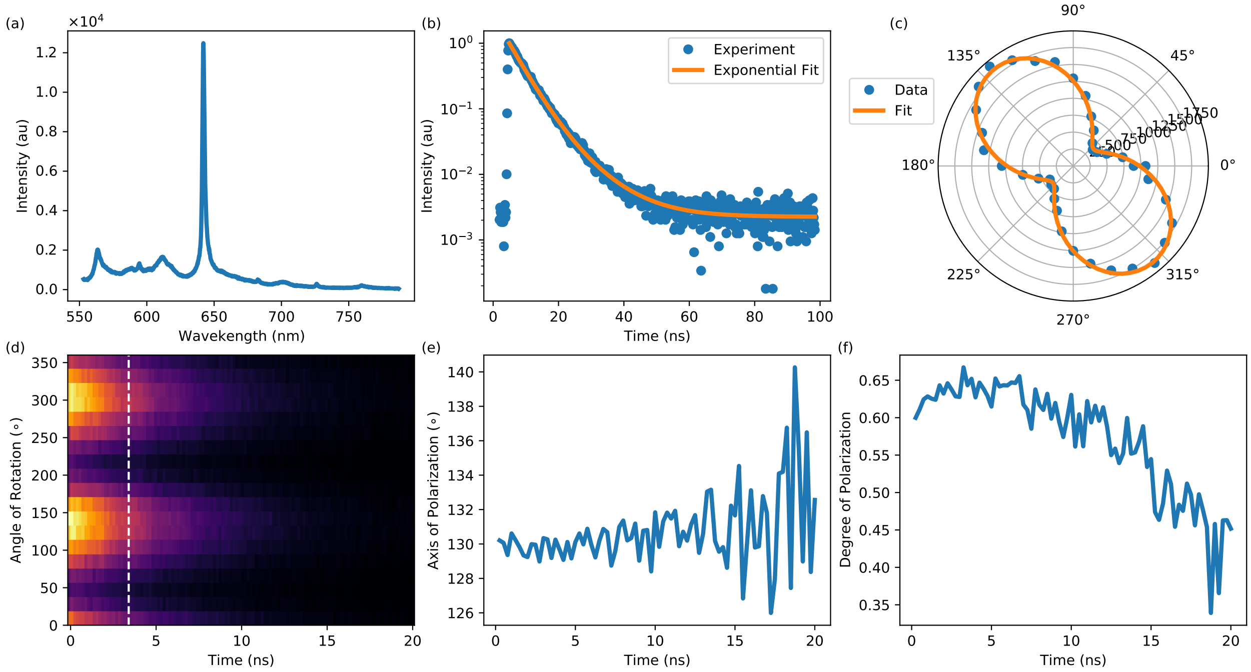

Figure S26: (a) Photoluminescence spectrum of an exemplary hBN nanoflake defect emitting at 642 nm, tagged as Emitter-3 in the main text. (b) Time-resolved PL intensity of Emitter-3 with a decay time of $\tau = 4.7$ ns extracted from the fit. (c) Emission polarization-dependent PL intensity, indicating a polarization angle of 130○, extracted from the fit function. (d) The PL intensity of Emitter-3 as a function of the rotation angle of the polarizer and decay time. (e) Axis of polarization as a function of decay time of the Emitter-3. (f) Visibility of linear degree of polarization as a function of decay time. For figures (e) and (f), 200 picosecond time bins are used.

S8 NV centers in diamond

Nitrogen-vacancy (NV) centers in diamond crystals are point defects composed of a substitutional nitrogen atom (N) and an adjacent vacant lattice site (V). The fluorescence spectrum of NV centers in diamond is characterized by a zero-phonon line (ZPL) at 637 nm, corresponding to the spin-triplet ground state ($^3A_2$) to the spin-triplet excited state (^3E) transition. In addition, the emission spectrum is recognized by a broad phonon side band emission from 637 nm to about 800 nm (see Fig. S27), corresponding to transitions from the spin-triplet excited state (^3E) to the short-lived vibronic states slightly above ground state ($^3A_2$) with added phonons, followed by a rapid decay to ground state[2, 3].

The emission of NV centers is bright and highly polarized, with a typical lifetime 12 ns in diamond crystals [4, 5]. The nanodiamonds containing few NV centers show longer lifetimes than NV centers in bulk diamonds due to reduced optical density of states (DOS), resulting from increased surface-volume ratio [6, 7, 8]. The nanodiamonds under investigation here (Adamas Nanotechnologies, 40 nm Carboxylated Red FND) have an average diameter of 40 nm and contain 1-4 single NV centers. The solution was dropcast onto a standard Si/SiO_2 chip and dried in ambient conditions. A fluorescence map of some nanodiamonds and the corresponding NV spectrum are shown in Fig. S27. The photophysical properties are shown in Fig. S28.

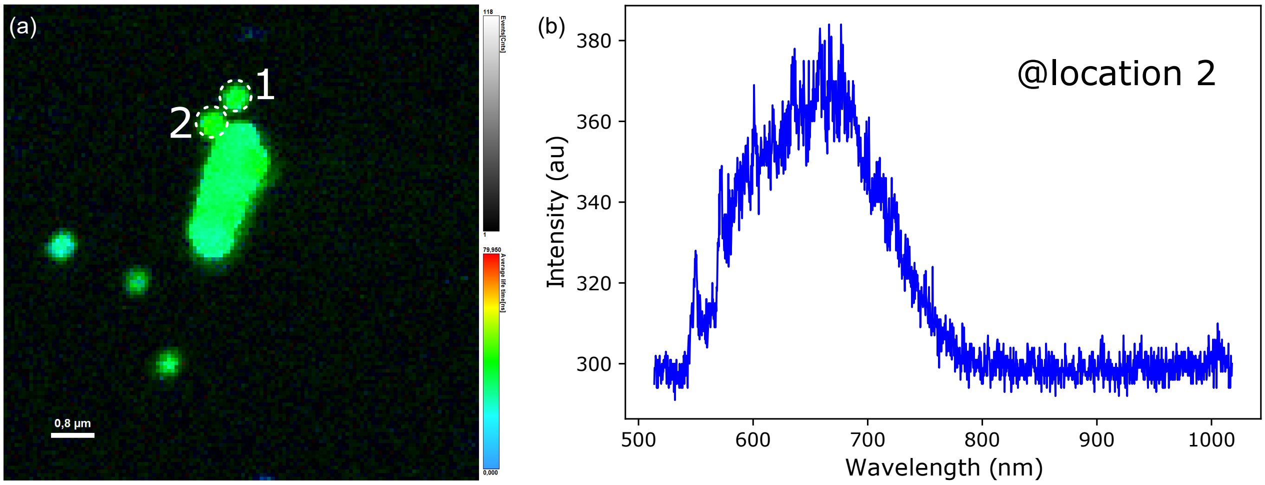

Figure S27: (a) PL of map of nanodiamond clusters obtained with an excitation laser wavelength of 530 nm. Also single nanodiamonds can be seen (by the diffraction limited spots. (b) A typical spectrum of NV centers in nanodiamond.

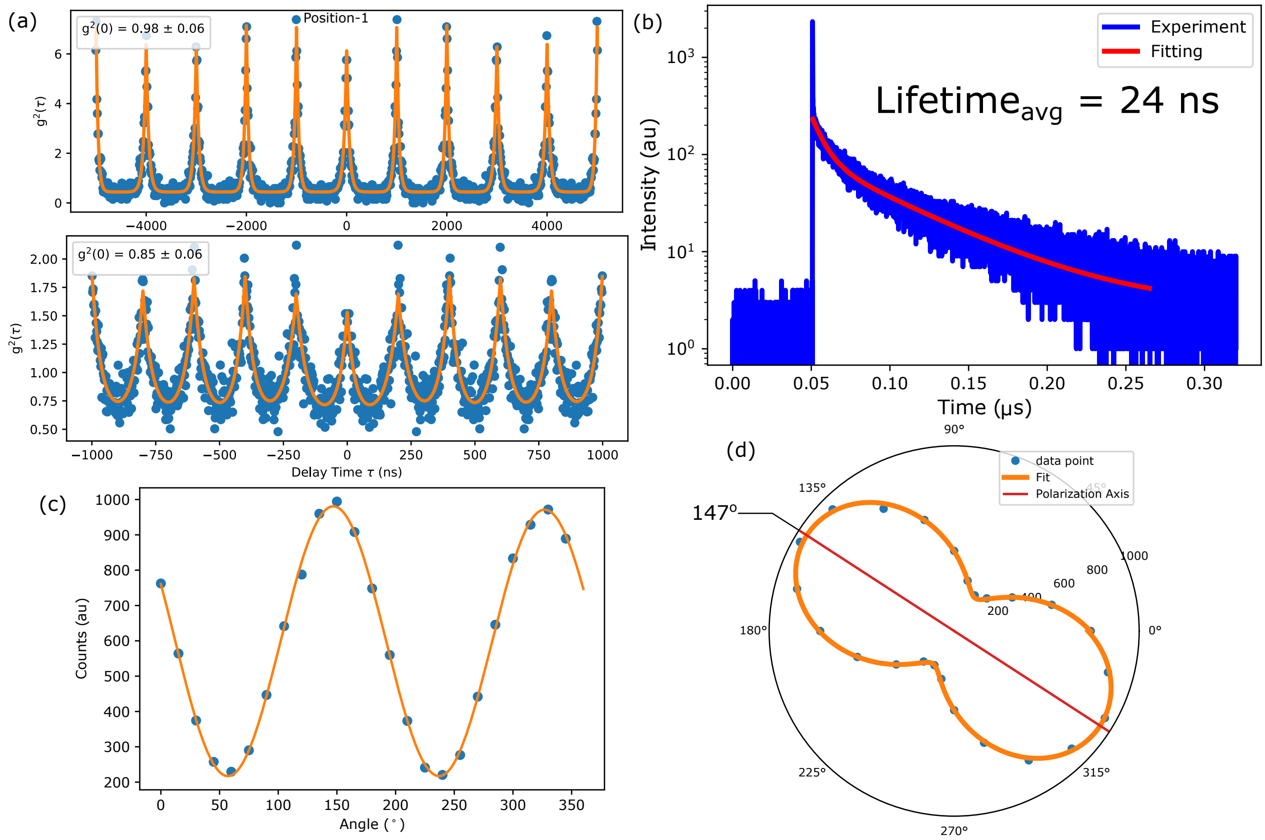

Figure S28: (a) The second-order correlation with $g^2(\tau = 0) = 0.9$, indicating that more than one NV center might be in this nanodiamond. (b) Typical lifetime of NV centers. A lifetime of 24 ns is extracted from a fit. (c) Linear and (d) polar plot of emission polarization extracted from time trace as mentioned before. With the excitation polarizer setting, a single NV can be selected (i.e., perpendicular to another NV center).

S9 Power-dependent temporal dynamics of polarization

To investigate the origin of this effect, we performed the previous investigation of temporal dynamics for different laser powers. However, no significant change is observed when the excitation laser power

is varied. Fig. S29 summarizes these results for four exemplary emitters.

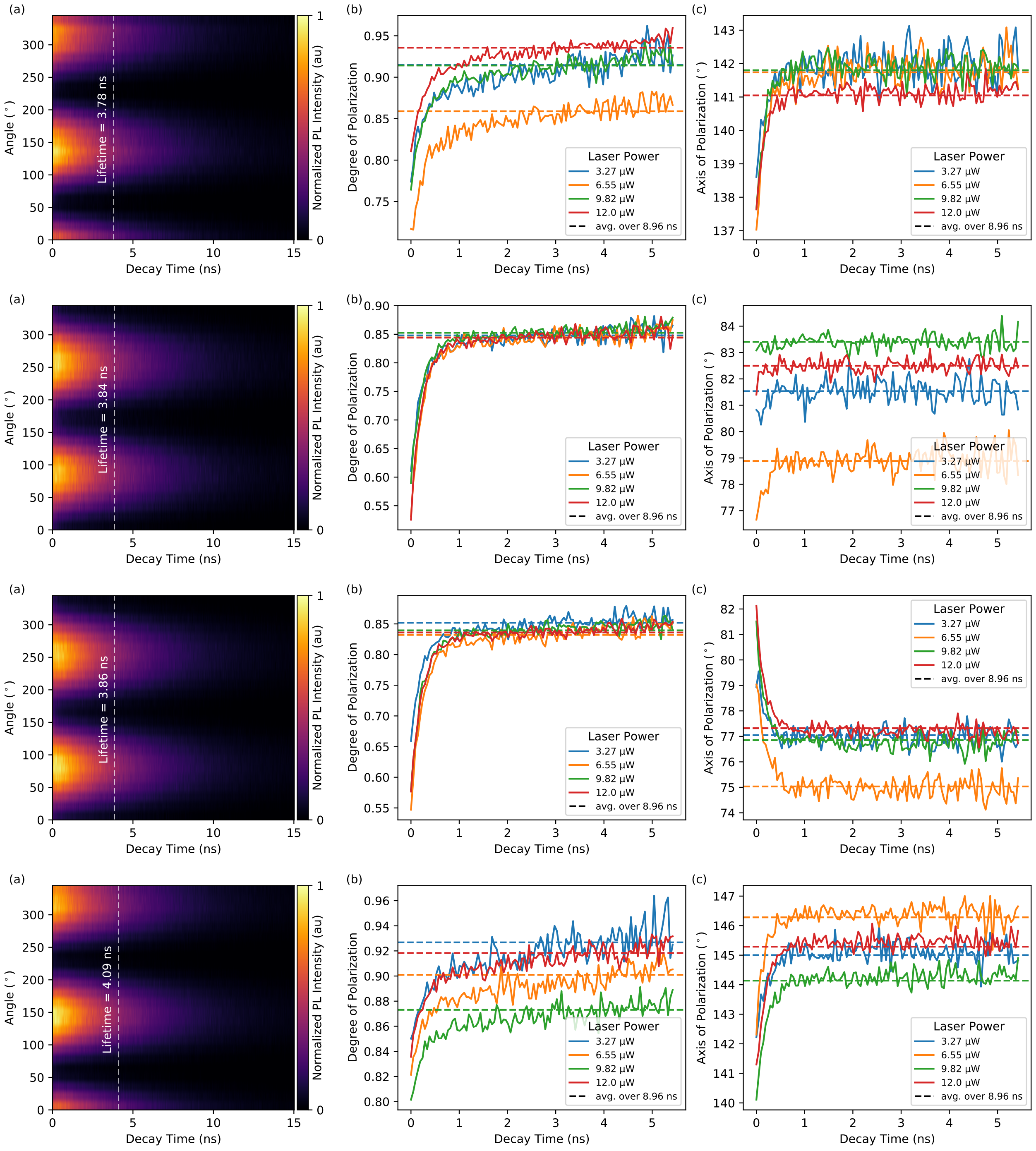

Figure S29: (a) Density plot of various emitter intensities (top to bottom) as function of the rotation angle and decay time for a specific laser power (b) Degree of polarization and (c) polarization axes of specific emitters (top to bottom) for different laser powers.

S10 DFT calculations

S10.1 Dipole calculations

The transition dipole moment (TDM) can be expressed as [9]

$$\mu = \frac{i\hbar}{(E_f - E_i)m} \langle \psi_f | \, \mathbf{p} \, | \psi_i \rangle \,, \tag{5}$$

where E_i and E_f are the eigenvalues of the initial and final orbitals, accordingly, m is the mass of an electron, and $\mathbf{p}$ is a momentum operator. It is important to note that the wave functions ψ_i and ψ_f in this case are taken from the different state configurations. Unlike the common TDM taken from the different Kohn-Sham orbitals under the same structural configuration, the excitation and emission mechanisms in hBN defects are the transition between the ground and excited states with different electron occupations, leading to different structural configurations. This can cause both wave functions to be significantly distinct. For the excitation TDM, we define ψ_i as the relaxed ground-state configuration, whilst ψ_f as the relaxed excited-state configuration. For the emission TDM, the roles of ψ_i and ψ_f are reversed. To extract the wave functions, the PyVaspwfc Python code was implemented [10]. As this code was written for calculating TDM from the wave function in the same configuration, we adapted the modified version and methodology outlined by Ref. [9] to handle the different wave function characteristics. Equivalently, the TDM can be expressed as

$$\mu = |\mu_x|\hat{x} + |\mu_y|\hat{y} + |\mu_z|\hat{z}, \tag{6}$$

where $\mu_z = 0$ indicates a purely *in-plane* TDM; otherwise, it has an *out-of-plane* component. As the excitation/emission axes are perpendicular to the dipoles, the dipole axes need to be rotated by 90° to make them compatible with the angle measured experimentally between the excitation/emission axes and the crystal axis. In addition, we project the dipole to the xy-plane to compare the visibility with the experiments.

S10.2 Dipoles of yellow emitter's candidates

The results for the C_2C_2-3 (for the nomenclature, see Sec. S10.4) defect is shown in the main text. According to Fig. 4 in the main text, from point 3, the emission can take place either directly to the ground state (point 1) or via another phonon mode (point 4). We have calculated both transition dipole moments for the ZPL (3−1 transition) and a (randomly chosen) phonon mode in the PSB (3−4 transition) and found only negligible difference in the relative angles (below 1°) as shown in Tab. S1 below. Hence, we restrict the analysis of emission dipole to the ZPL channel (transition from points 3 to 1).

Table S1: Emission decay pathway of C_2C_2-3

Properties	Points 3 to 1	Points 3 to 4
Emission wavelength (nm)	573	708
Emission dipole (°)	17.9	18.6
Emission polarization (°)	12.1	11.4
Dipole misalignment (°)	1.1	0.3
Polarization misalignment (°)	1.0	0.3

Among the 126 investigated defects, we identified two more promising candidates namely, the C_NV_N (see Fig. S30) C_2C_2-5 (see Fig. S31) defects. As outlined in the main text, we believe that the C_2C_2-3 defect is the most likely candidate after taking all data altogether into account. The results of the calculations of all 126 defects are presented in the attached Excel spreadsheet and can be accessed via `https://doi.org/10.5281/zenodo.10288562` (note: will be filled after review).

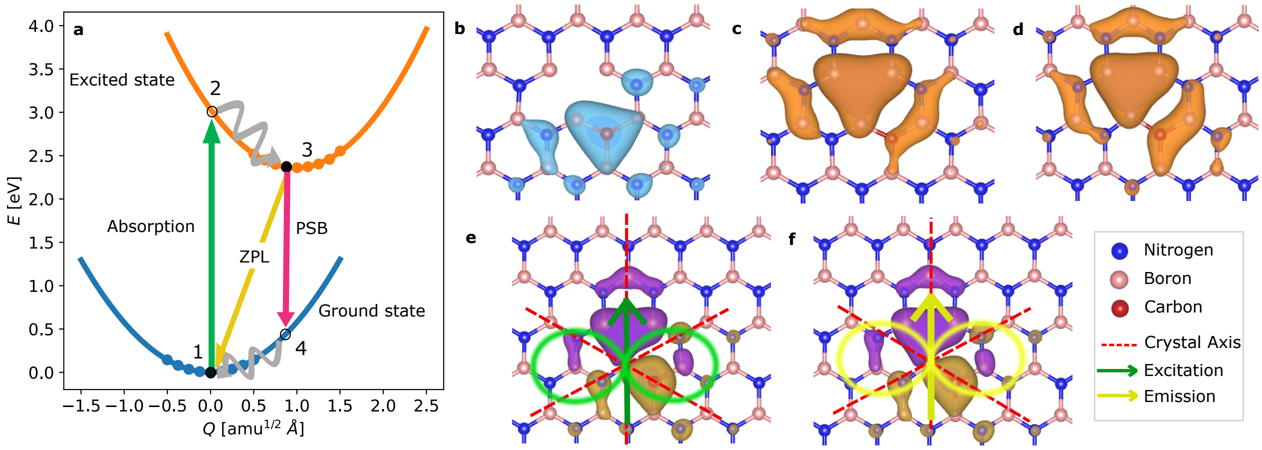

Figure S30: (a) Potential energy surface of a C_NV_N defect representing the complete excitation and emission process, consisting of the absorption (green line), the zero-phonon line (ZPL, yellow line, here 525 nm), and the phonon sideband (PSB, magenta line). (b)-(d) The probability density $|\psi|^2$ of electron occupations in ground state at point 1 and excited states at points 2 and 3, respectively. (e) The charge difference between points 1 and 2 is shown by the isosurfaces, where the green arrow indicates the excitation dipole axis with light radiation in green shade. The excitation axis makes 29.8° relative to the crystal axis (red dashed line). (f) The charge difference between points 1 and 3 with the yellow arrow indicating the emission dipole axis with light radiation in yellow shade. The emission axis makes 28.1° relative to the crystal axis.

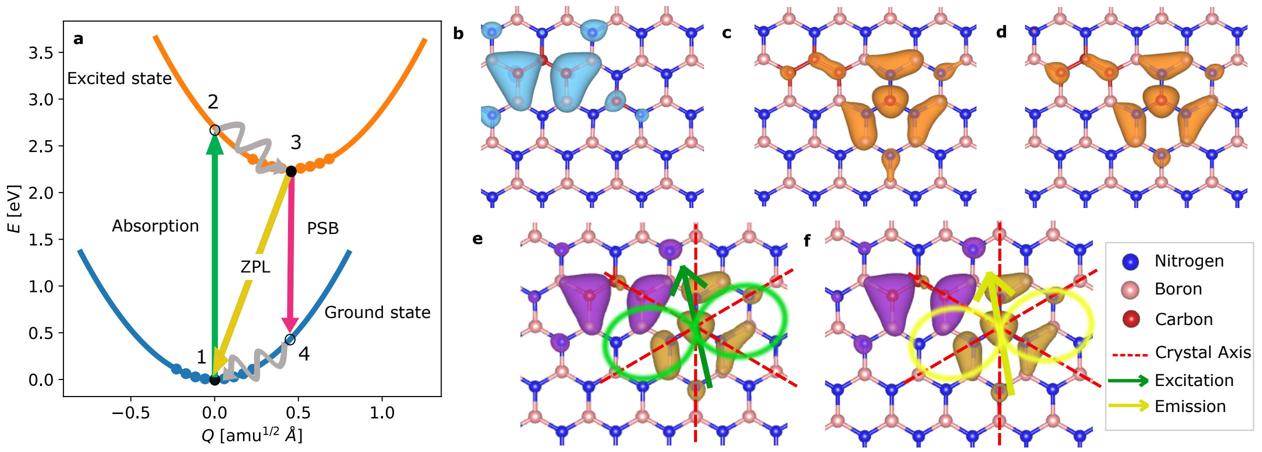

Figure S31: (a) Potential energy surface of a C_2C_2-5 defect representing the complete excitation and emission process, consisting of the absorption (green line), the zero-phonon line (ZPL, yellow line, here 562 nm), and the phonon sideband (PSB, magenta line). (b)-(d) The probability density $|\psi|^2$ of electron occupations in ground state at point 1 and excited states at points 2 and 3, respectively. (e) The charge difference between points 1 and 2 is shown by the isosurfaces, where the green arrow indicates the excitation dipole axis with light radiation in green shade. The excitation axis makes 12.3° relative to the crystal axis (red dashed line). (f) The charge difference between points 1 and 3 with the yellow arrow indicating the emission dipole axis with light radiation in yellow shade. The emission axis makes 13.7° relative to the crystal axis.

S10.3 Charged-state defects

In this section, we summarize the angles of excitation/emission axes with respect to the nearest crystal axis regardless of their (charge-state) stability (see Fig. S32, S33, and S34). We emphasize that we aim first to explore the defects exhibiting similar excitation/emission properties as those obtained in the experiment. We then rule out some defects based on zero-phonon line, the relative dipole orientations, and the linear in-plane polarization visibility as discussed in the main text. Subsequently, we need to investigate only promising defects for charge-state stability, which is presented in Sec. S10.5. The complete data-set is also available into a Excel spreadsheet via link: `https://doi.org/10.5281/zenodo.10288562`

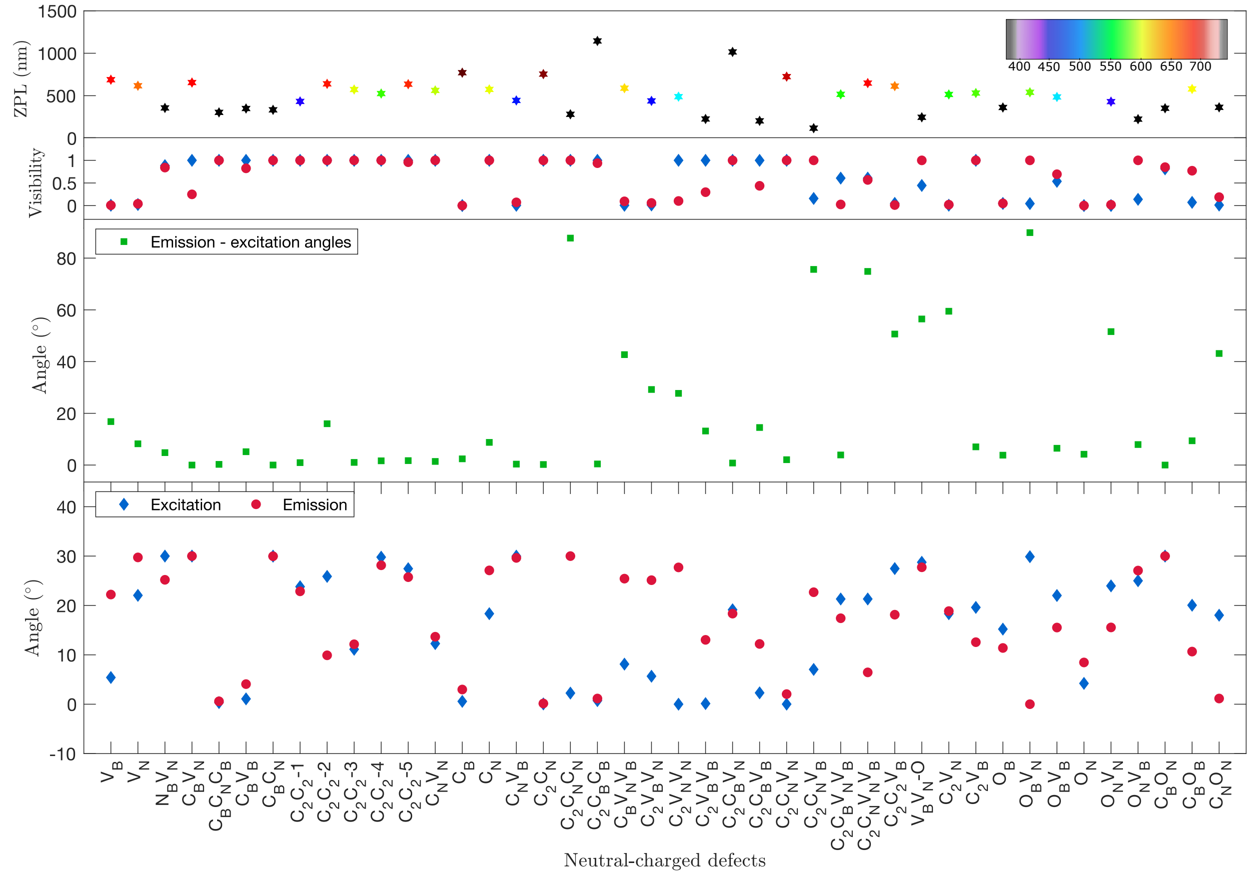

Figure S32: Theoretical characterization of 42 neutral-charged defects, where the topmost panel indicates the zero phonon line (ZPL) represented by the color. The black dots represent the ZPLs which are out of visible light. The second top panel depicts the linear in-plane polarization visibility of excitation (blue) and emission (red); the middle panel illustrates the misalignment between excitation and emission; and the bottom panel shows the angle of excitation (blue) and emission (red) with respect to the nearest crystal axis.

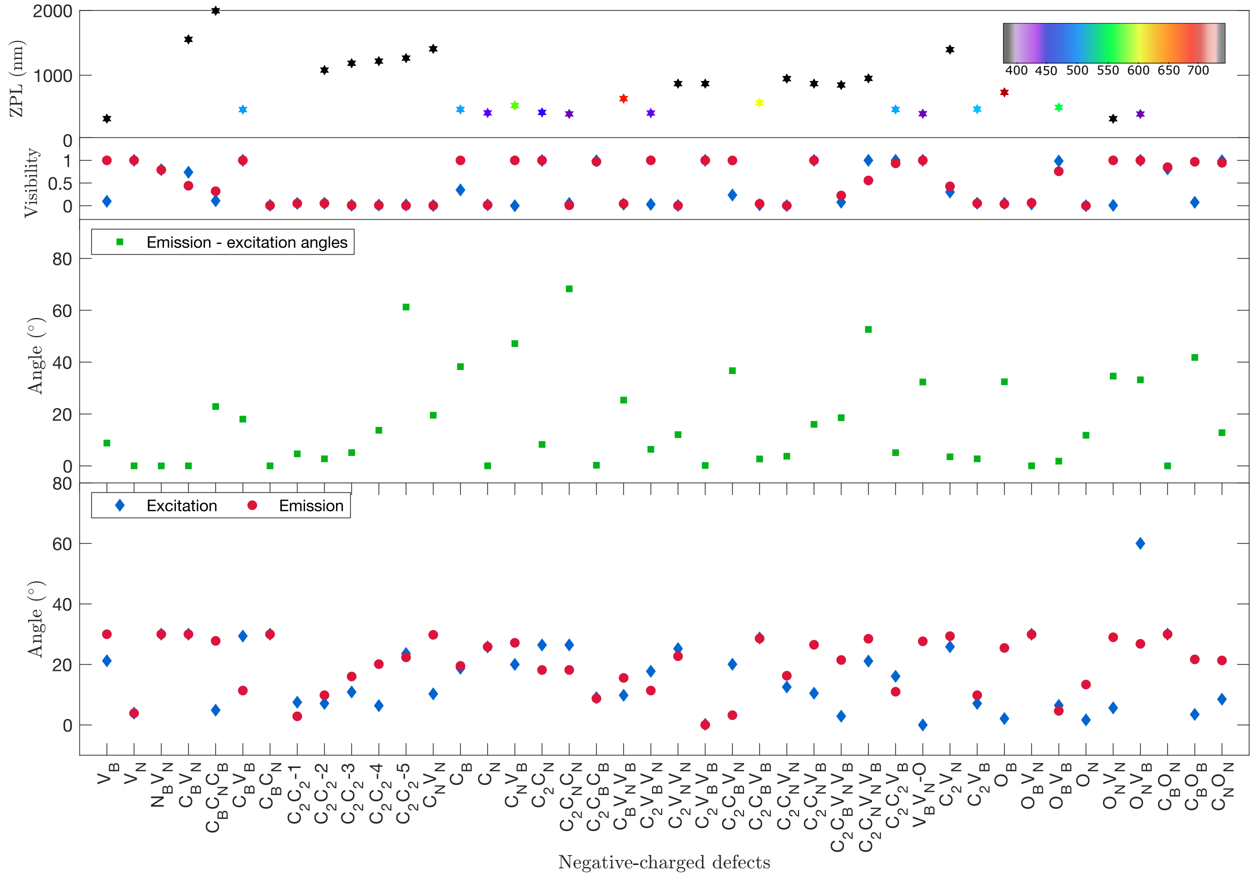

Figure S33: Theoretical characterization of 42 single negatively-charged defects , where the topmost panel indicates the zero phonon line (ZPL) represented by the color. The black dots represent the ZPLs which are out of visible light. The second top panel depicts the linear in-plane polarization visibility of excitation (blue) and emission (red); the middle panel illustrates the misalignment between excitation and emission; and the bottom panel shows the angle of excitation (blue) and emission (red) with respect to the nearest crystal axis.

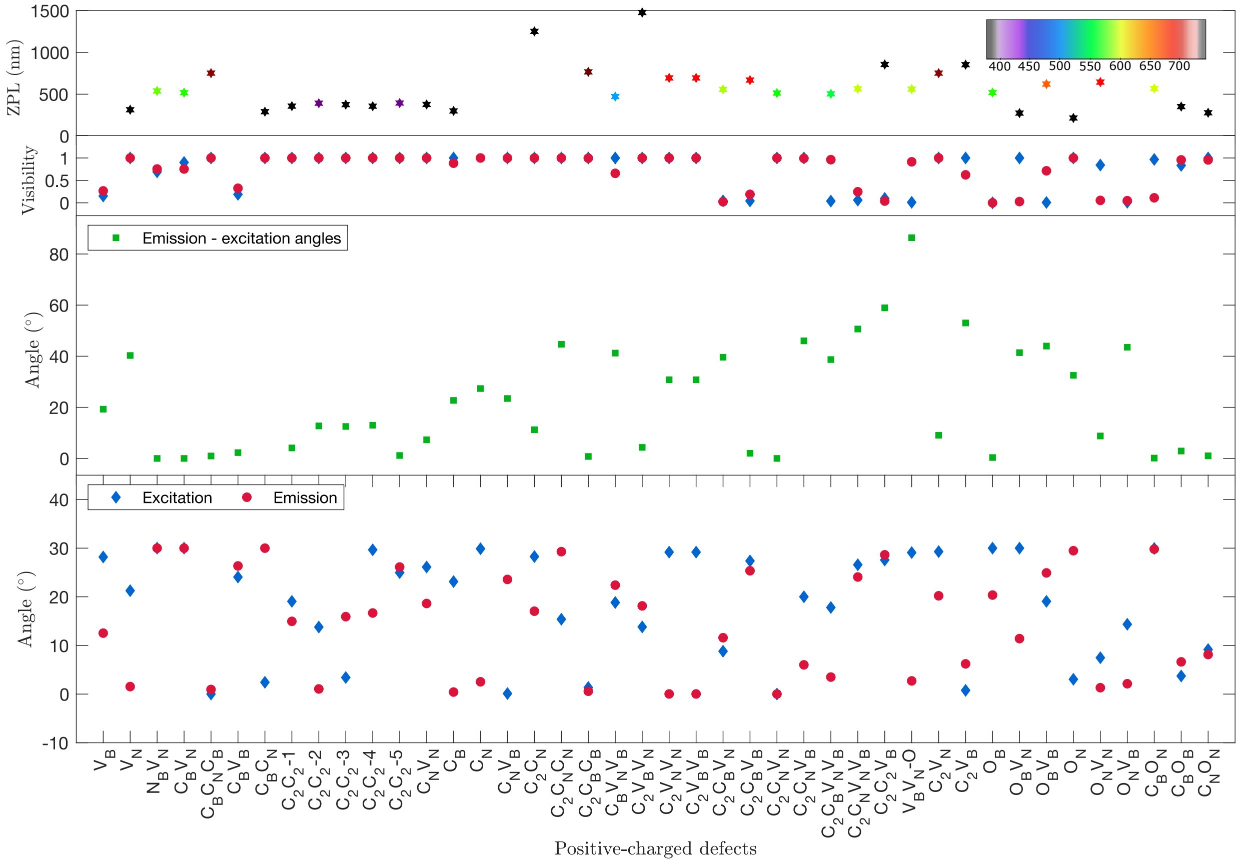

Figure S34: Theoretical characterization of 42 singly positive-charged defects, where the topmost panel indicates the zero phonon line (ZPL) represented by the color. The black dots represent the ZPLs which are out of visible light. The second top panel depicts the linear in-plane polarization visibility of excitation (blue) and emission (red); the middle panel illustrates the misalignment between excitation and emission; and the bottom panel shows the angle of excitation (blue) and emission (red) with respect to the nearest crystal axis.

S10.4 C_2C_2 configurations

As different configurations of the C_2C_2 complex are possible, we identify them by a number. These are illustrated for exemplary configurations in Fig. S35.

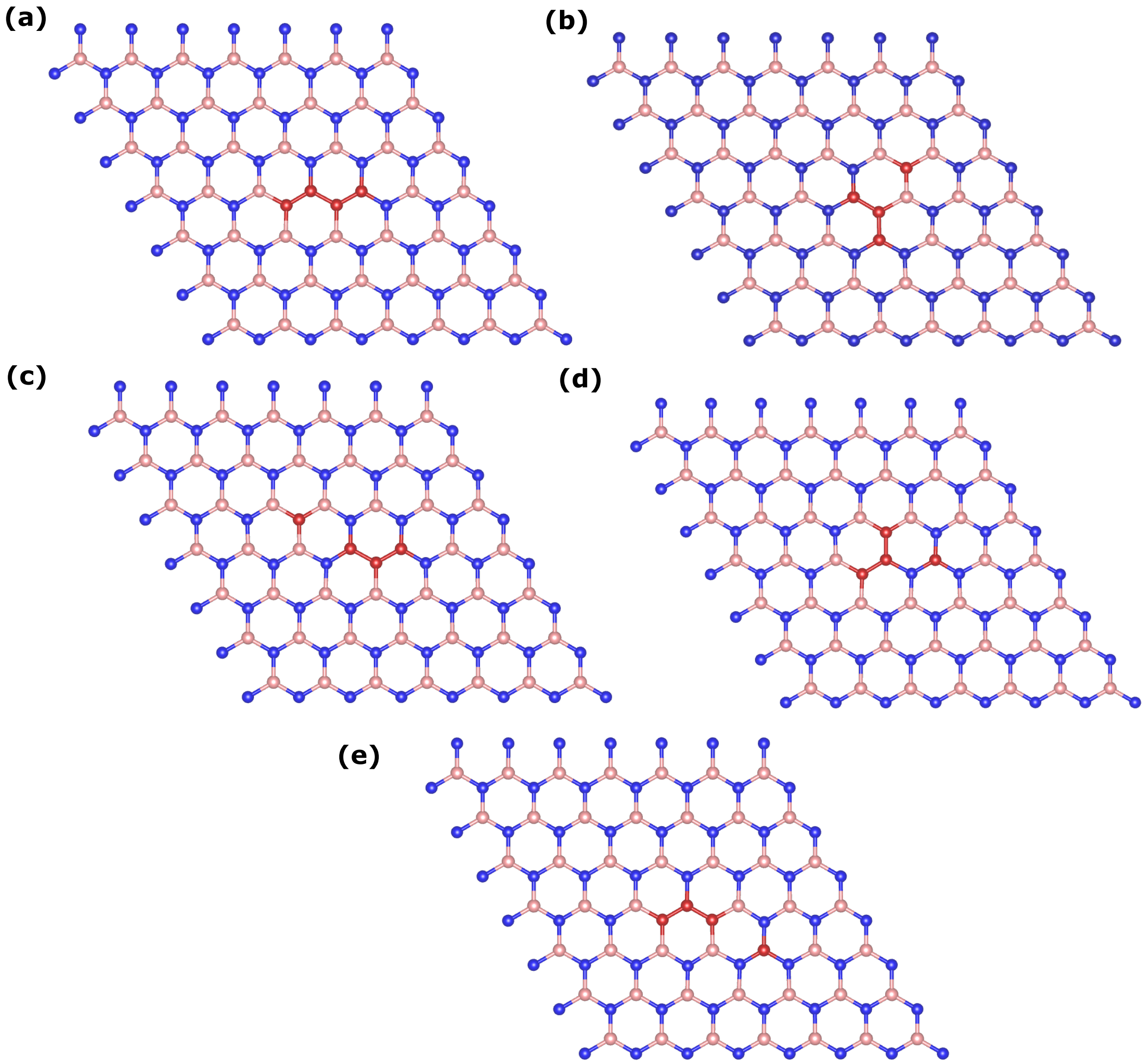

Figure S35: Structural configurations of C_2C_2 defects where (a) four carbon atoms are bonded together (C_2C_2-1); (b) three carbon atoms are bonded and one carbon atom is isolated by a boron atom (C_2C_2-2); (c) similar to (b) but with different orientation (C_2C_2-3); (d) three carbon atoms are bonded and one carbon atom is isolated by a nitrogen atom (C_2C_2-4); (e) same as (d) but with different orientation (C_2C_2-5). Note that for the C_2C_2-3 and C_2C_2-5 configurations, we also investigated the other five configurations obtained from the rotations of such defect (not shown here).

S10.5 Defect formation energy

The promising defect candidates for being responsible for the yellow emission are C_2C_2-3, C_2C_2-5, and C_NV_N. Here, the defect formation energy, denoted by $E^f[D^q]$, has been computed to determine the stability of these defects:

$$E^f[D^q] = E_{\mathrm{tot}}[D^q] - E_{\mathrm{tot}}[\mathrm{hBN}] - \sum_i n_i \mu_i + q(E_{\mathrm{vbm}} + E_{\mathrm{fermi}}), \tag{7}$$

where $E_{\mathrm{tot}}[D^q]$ is the total energy of hBN with a defect; $E_{\mathrm{tot}}[\mathrm{hBN}]$ is the total energy of pristine hBN; n_i is the number of removed or added defects; μ_i is a chemical potential of an isolated element; q is the charge of a defect; E_{vbm} is the energy at valence band maximum; and E_{fermi} is Fermi energy. Theoretically, a C_2C_2 defect was found to be feasible to form a stable defect (see Fig. S36).

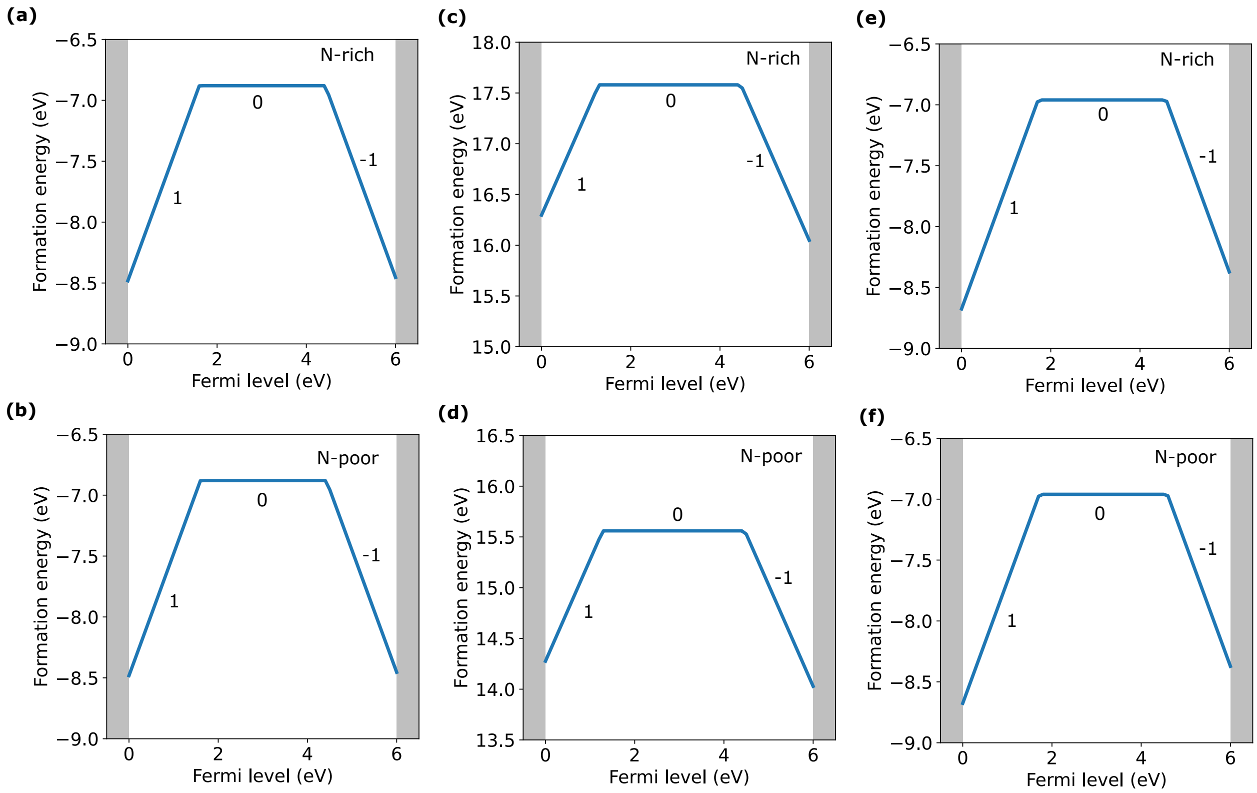

Figure S36: Defect formation energy where (a) and (b) are for C_2C_2-3; (c) and (d) are for C_NV_N; and (e) and (f) are for C_2C_2-5.

S10.6 Effects of an electric field on dipole orientation

We applied the out-of-plane electric field to investigate the behavior of the dipoles. This is to mimic the experimentally observed temporal dynamics. The applied fields were up a strength of 0.7 V/Å. To put into some perspective, the electric field magnitude of about 0.7 V/Å corresponds to that from a single electron as a point charge sitting either above or below the defect by a few angstroms (i.e., in the neighboring layer). This field strength is also similar to the one realized in an hBN experiment involving Stark tuning and was shown to have an impact on the emitters (even though no polarization was studied) [11]. The results for the impact on polarization for the promising defects are shown in Fig. S37.

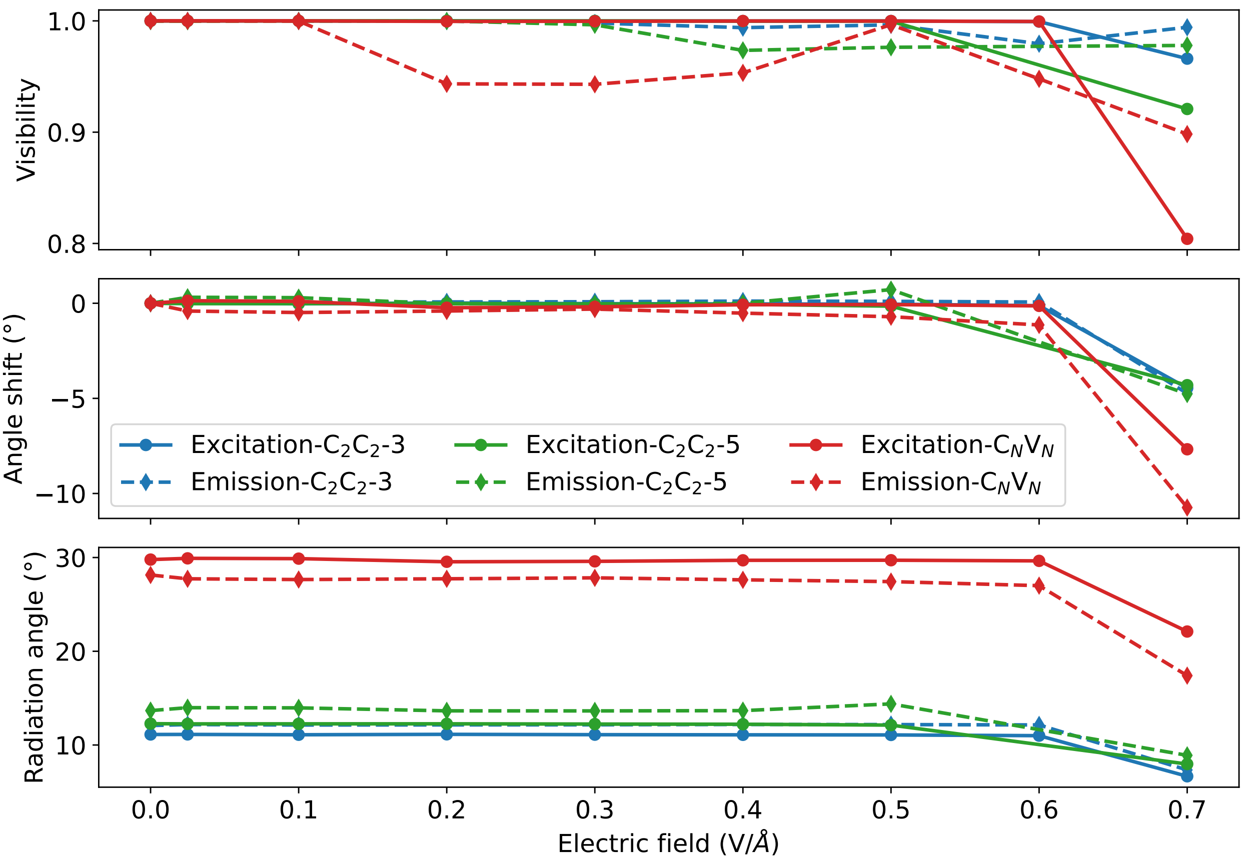

Figure S37: Variation of excitation and emission axes affected by an out-of-plane electric field. The solid line represents the excitation, whereas the dotted line represents the emission. (a) The linear in-plane polarization visibility, (b) the angle of excitation/emission axes shifted from that with the absence of electric field, and (c) the excitation/emission angle measured with respect to the nearest crystal axis.

S10.7 Effect of strain on dipole orientation

Similar to the previous section, we also studied the effects of strain on the polarization. The applied bi-axial strain was on the order of ±1% (see Fig. S38).

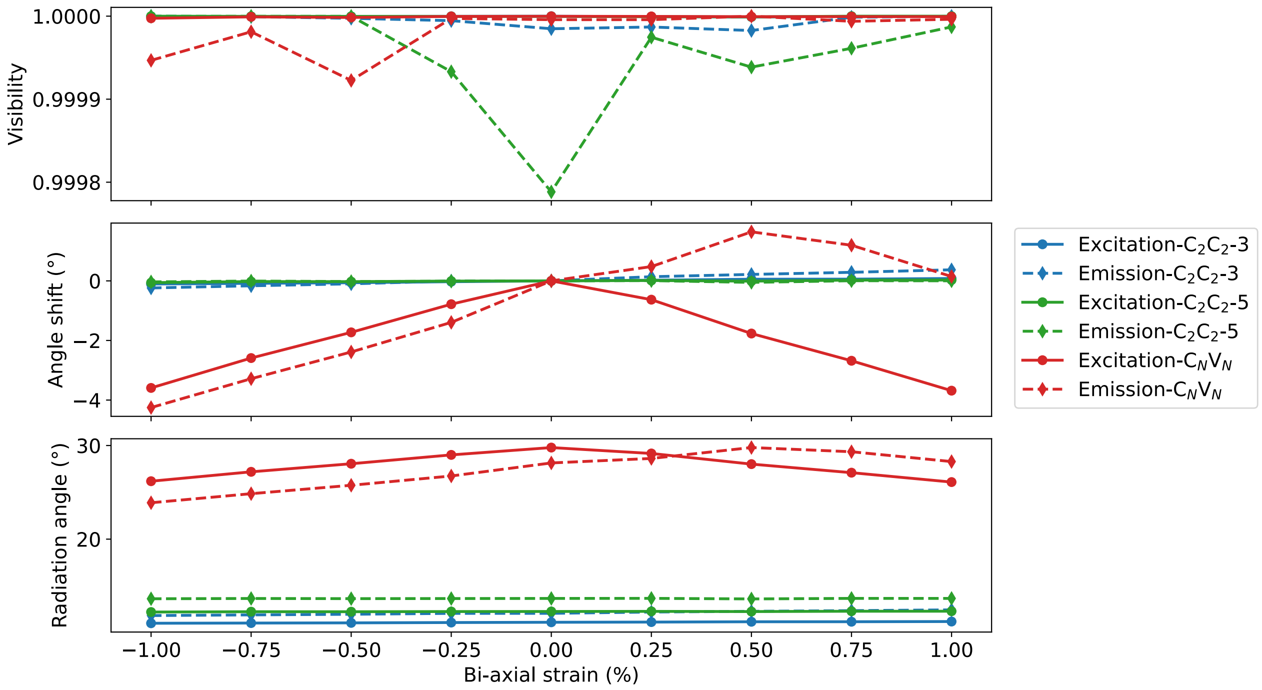

Figure S38: Variation of excitation and emission axes affected by bi-axial strain. The solid line represents the excitation, whereas the dotted line represents the emission. (a) The linear in-plane polarization visibility, (b) the angle of excitation/emission axes shifted from that with the unstrained condition, and (c) the excitation/emission angle measured with respect to the nearest crystal axis.

S10.8 Effect of strain on ZPL

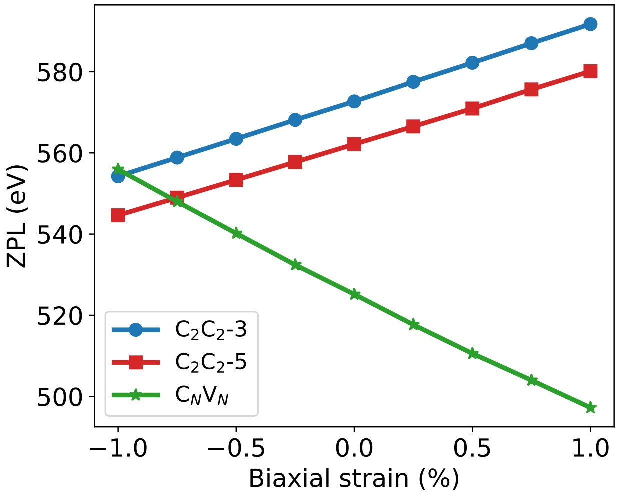

Figure S39: Variation of ZPL affected by a biaxial strain.

In this section, we investigated how the strain changes the ZPL of each defect. Fig. S39 illustrates that the strain can shift the ZPL of all defects by ±20 nm for ±1% of strain. This is also consistent with our previous study quantifying the ZPL variation due to strain for the defect with in-plane deformation [12].

References

[1] Anand Kumar, Chanaprom Cholsuk, Ashkan Zand, Mohammad N. Mishuk, Tjorben Matthes, Falk Eilenberger, Sujin Suwanna, and Tobias Vogl. Localized creation of yellow single photon emitting carbon complexes in hexagonal boron nitride. *APL Mater.*, 11(7):071108, 07 2023. `doi:10.1063/5.0147560`.

[2] Dominik B Bucher, Diana PL Aude Craik, Mikael P Backlund, Matthew J Turner, Oren Ben Dor, David R Glenn, and Ronald L Walsworth. Quantum diamond spectrometer for nanoscale NMR and ESR spectroscopy. *Nat. Protoc.*, 14(9):2707–2747, 2019.

[3] Marcus W Doherty, Neil B Manson, Paul Delaney, Fedor Jelezko, Jörg Wrachtrup, and Lloyd CL Hollenberg. The nitrogen-vacancy colour centre in diamond. *Phys. Reps*, 528(1):1–45, 2013.

[4] AT Collins, MF Thomaz, and Maria Isabel B Jorge. Luminescence decay time of the 1.945 eV centre in type Ib diamond. *J. Phys. C: Solid State Phys.*, 16(11):2177, 1983.

[5] A Batalov, C Zierl, T Gaebel, P Neumann, I-Y Chan, G Balasubramanian, PR Hemmer, F Jelezko, and J Wrachtrup. Temporal coherence of photons emitted by single nitrogen-vacancy defect centers in diamond using optical rabi-oscillations. *Phys. Rev. Lett.*, 100(7):077401, 2008.

[6] Justus Christinck, Beatrice Rodiek, Marco López, Helmuth Hofer, Hristina Georgieva, and Stefan Kück. Characterization of the angular-dependent emission of nitrogen-vacancy centers in nanodiamond. *Appl. Phys. B*, 126:161, 2020.

[7] Jonas Gutsche, Ashkan Zand, Marek Bültel, and Artur Widera. Revealing superradiant emission in the single-to-bulk transition of quantum emitters in nanodiamond agglomerates. *New J. Phys.*, 24(5):053039, 2022.

[8] Jelle Storteboom, Philip Dolan, Stefania Castelletto, Xiangping Li, and Min Gu. Lifetime investigation of single nitrogen vacancy centres in nanodiamonds. *Opt. Express*, 23(9):11327–11333, 2015.

[9] Joel Davidsson. Theoretical polarization of zero phonon lines in point defects. *J. Phys.: Condens. Matter*, 32:385502, 9 2020.

[10] Liming Liu. PyVaspwfc. https://github.com/liming-liu/pyvaspwfc, 2017, (accessed October 1, 2022). URL: `https://github.com/liming-liu/pyvaspwfc`.

[11] Ivan Zhigulin, Jake Horder, Viktor Ivády, Simon J.U. White, Angus Gale, Chi Li, Charlene J. Lobo, Milos Toth, Igor Aharonovich, and Mehran Kianinia. Stark effect of blue quantum emitters in hexagonal boron nitride. *Phys. Rev. Appl.*, 19:044011, Apr 2023. URL: `https://link.aps.org/doi/10.1103/PhysRevApplied.19.044011`, `doi:10.1103/PhysRevApplied.19.044011`.

[12] Chanaprom Cholsuk, Sujin Suwanna, and Tobias Vogl. Tailoring the emission wavelength of color centers in hexagonal boron nitride for quantum applications. *Nanomaterials*, 12:2427, 7 2022. URL: `https://www.mdpi.com/2079-4991/12/14/2427`, `doi:10.3390/nano12142427`.
